# NanoGalaxy: Nanopore long-read sequencing data analysis in Galaxy

**DOI:** 10.1093/gigascience/giaa105

**Published:** 2020-10-17

**Authors:** Willem de Koning, Milad Miladi, Saskia Hiltemann, Astrid Heikema, John P Hays, Stephan Flemming, Marius van den Beek, Dana A Mustafa, Rolf Backofen, Björn Grüning, Andrew P Stubbs

**Affiliations:** Department of Pathology, Clinical Bioinformatics Unit, Erasmus University Medical Centre, Wytemaweg 80, 3015 CN, Rotterdam, the Netherlands; Department of Pathology, Tumor Immuno-Pathology Laboratory, Erasmus University Medical Centre, 's Gravendijkwal 230, 3015 CE, Rotterdam, the Netherlands; Department of Computer Science, Bioinformatics Group, University of Freiburg, 79110 Freiburg im Breisgau, Germany; Department of Pathology, Clinical Bioinformatics Unit, Erasmus University Medical Centre, Wytemaweg 80, 3015 CN, Rotterdam, the Netherlands; Department of Medical Microbiology and Infectious Diseases, Erasmus University Medical Centre, 's Gravendijkwal 230, 3015 CE, Rotterdam, the Netherlands; Department of Medical Microbiology and Infectious Diseases, Erasmus University Medical Centre, 's Gravendijkwal 230, 3015 CE, Rotterdam, the Netherlands; Department of Computer Science, Bioinformatics Group, University of Freiburg, 79110 Freiburg im Breisgau, Germany; Department of Stem Cells and Tissue Homeostasis, Institut Curie, PSL Research University, 75005 Paris, France; Department of Pathology, Tumor Immuno-Pathology Laboratory, Erasmus University Medical Centre, 's Gravendijkwal 230, 3015 CE, Rotterdam, the Netherlands; Department of Computer Science, Bioinformatics Group, University of Freiburg, 79110 Freiburg im Breisgau, Germany; Department of Computer Science, Bioinformatics Group, University of Freiburg, 79110 Freiburg im Breisgau, Germany; Department of Pathology, Clinical Bioinformatics Unit, Erasmus University Medical Centre, Wytemaweg 80, 3015 CN, Rotterdam, the Netherlands

**Keywords:** long-read sequencing, Nanopore, Galaxy, reproducibility, workflows

## Abstract

**Background:**

Long-read sequencing can be applied to generate very long contigs and even completely assembled genomes at relatively low cost and with minimal sample preparation. As a result, long-read sequencing platforms are becoming more popular. In this respect, the Oxford Nanopore Technologies–based long-read sequencing “nanopore" platform is becoming a widely used tool with a broad range of applications and end-users. However, the need to explore and manipulate the complex data generated by long-read sequencing platforms necessitates accompanying specialized bioinformatics platforms and tools to process the long-read data correctly. Importantly, such tools should additionally help democratize bioinformatics analysis by enabling easy access and ease-of-use solutions for researchers.

**Results:**

The Galaxy platform provides a user-friendly interface to computational command line–based tools, handles the software dependencies, and provides refined workflows. The users do not have to possess programming experience or extended computer skills. The interface enables researchers to perform powerful bioinformatics analysis, including the assembly and analysis of short- or long-read sequence data. The newly developed “NanoGalaxy" is a Galaxy-based toolkit for analysing long-read sequencing data, which is suitable for diverse applications, including *de novo* genome assembly from genomic, metagenomic, and plasmid sequence reads.

**Conclusions:**

A range of best-practice tools and workflows for long-read sequence genome assembly has been integrated into a NanoGalaxy platform to facilitate easy access and use of bioinformatics tools for researchers. NanoGalaxy is freely available at the European Galaxy server https://nanopore.usegalaxy.eu with supporting self-learning training material available at https://training.galaxyproject.org.

## Background

Short-read sequencing has become a routine technique within clinical diagnostics [1]. However, the short length of the reads obtained (150–300 bp) complicates the assembly of genomes, especially for highly repetitive regions and the detection of structural variation [2–4]. Furthermore, even “state-of-the-art” algorithms cannot overcome the issues associated with genome mapping or assembly using short-read sequences. Importantly, advances in sequencing technology now allow “long-read sequencing" to be performed. The 2 prominent long-read sequencing platforms are nanopore sequencing by Oxford Nanopore Technologies and single-molecule real-time sequencing by Pacific Biosciences [5,6]. These platforms generate sequence reads much longer than those of the classic short-read technologies, including long reads from single DNA molecules and without the need of PCR amplification (>10 kb on average). Moreover, utilizing these technologies, library preparation and sequencing may be performed outside of traditional research laboratories, with sequencing outputs generated in real time [7]. Protocols that require no PCR amplification also permit the direct detection of base modifications [8].

Analyzing the large amount of data generated by the short- and long-read sequencing technologies is a complex, multi-step process that is computationally intensive and often requires bioinformatics expertise. Specifically, for each step in the analysis, a set of different tools or software may be needed. For example, *de novo* assembly is performed via a combination of multiple alignments, assembly and polishing tools, each utilizing its own input parameters. Such tools are typically executed from a UNIX command line and require extensive computational resources, adding to the complexity of the analysis process. Command line–based workflow managers such as Snakemake and Nextflow [9,10] can be used for analysing the data. However, these solutions require having expertise in working from the command line. On the other side, some web-based solutions have also been offered. For example the EPI2ME platform offers a cloud-based solution with a web interface. The platform supports practical solutions for a limited set of application scenarios and provides a limited flexibility for configuring the underlying workflows. Here, the Galaxy platform offers a flexible data analysis platform with a high degree of flexibility, similar to the command line–based workflow managers, and an accessible web interface.

The Galaxy platform reduces the data analysis complexity and implements a standardized and user-friendly interface that accommodates command line tools and refined workflows complete with their dependencies [11]. The platform hosts a wide range of tools/software and is widely used for bioinformatics analysis within the biological science community [12,13]. Here we introduce the NanoGalaxy toolkit for analysing Nanopore long-read data. NanoGalaxy comprises a series of integrated Galaxy-based tools that enable researchers to generate powerful short- or long-sequence read assemblies for genomic and plasmid bioinformatics analyses. The NanoGalaxy toolkit is a user-friendly environment that can be utilized inside or outside of traditional research laboratories.

## Findings

### Tools

We have integrated a large collection of long-read sequence tools into the Galaxy platform, the NanoGalaxy toolkit, including diverse applications for the analyses of long-read sequences (Table 1). This toolkit is freely available from the Galaxy ToolShed and has additionally been made available as a specialized GalaxyEU subdomain (https://nanopore.usegalaxy.eu).

**Table 1:** NanoGalaxy toolkit

Category	Tool name
*De novo* genome assembly	● Flye [14]● Canu [15]● Unicycler [16]● Wtdbg2 [17]● Miniasm [18]● Racon [19]● Spades [20]● Medaka (2 tools) [21]
Long-read mapping	● Minimap2 [22]
	● GraphMap (2 tools) [23]
Polishing, quality control, and pre-processing	● ont_fast5_api (4 tools) [24]● Nanopolish (3 tools) [25]● Porechop [26]● Filtlong [27]● Poretools (13 tools) [28]● Pilon [29]
Visualization	● Nanoplot [30]
	● Bandage (2 tools) [31]
	● Circos [32]
Taxonomy and metagenomics	● Kraken2 [33]
	● PlasFlow [34]
	● Staramr [35]
Methylation	● Nanopolish (1 tool) [25]
Variant calling	● Medaka (2 tools) [21]

### Workflows

To increase the utility of this toolkit, we have developed a set of Galaxy workflows performing common analysis tasks using the tools in the NanoGalaxy toolkit.

#### Metagenomics taxonomic classification

The base quality of nanopore sequencing reads is constantly improving, making the actual assembly of reads more reliable. Furthermore, the long reads generated by nanopore sequencing can be used to provide valuable information from metagenomics data, including taxonomic classifications.

Kraken2 is a *k*-mer–based classification technique that can efficiently assign the taxa of long reads that are resilient to the noisy nature of long-read data. The input reads for Kraken2 are compared to a database containing different classes and domains of life that are pre-indexed for algorithm efficiency. Within the NanoGalaxy toolkit we provide a workflow for taxonomic classification using Kraken2, including the post-processing of data and visualization of the results as interactive pie charts using the Krona tool [36].

#### Nanopolish tutorials

Nanopolish includes an extensive set of software tools for analysing nanopore long-read information at the raw signal level. Furthermore, accompanying Nanopolish documentation provides intuitive tutorials on common scenarios, such as variation analysis and base methylation calling from the raw and mapped signals [25]. We have integrated Nanopolish and its tutorials into NanoGalaxy in the form of workflows that can be used by researchers to analyse and interpret common quality values for their data.

#### De novo assembly of genome with highly repetitive repeats

Compared to short reads, long-read data have the advantage of facilitating the assembly of large genomes that contain high numbers of repetitive elements. Schmid et al. utilized Flye and several other tools to generate a comprehensive assembly of the *Pseudomonas koreensis* genome, identifying that the genome has near identical repeat pairs up to 70 kb in length [37]. These workflows have also been integrated in the NanoGalaxy toolkit.

### Worked example: Antimicrobial resistance

As a further illustration of the utility of the NanoGalaxy toolkit and workflows, we describe below a full end-to-end workflow within Galaxy. This analysis pipeline performs a microbial resistance detection in clinical samples. We describe this workflow in more detail in our training manual on the Galaxy Training materials repository (https://training.galaxyproject.org; Antibiotic resistance detection).

#### Background

According to the World Health Organization and the Organisation for Economic Co-operation and Development, antimicrobial resistance (AMR) has become one of the biggest threats to global health, food security, and economic development [38, 39]. Approximately 50,000 lives per year are lost due to AMR infections within the USA and Europe [40], and AMR infections are expected to increase, reaching 10 million deaths per year by 2050 [40].

Furthermore, the misuse of antibiotics in the medical, veterinary, and agricultural sectors continues to contribute to the alarming global increase in antibiotic-resistant infections—an increase that may ultimately lead to an era where common infections could once again be lethal. However, the (rapid) detection of AMR pathogens and their resistances in diseases, food, and the environment are pillars by which increasing AMR could be detected, monitored, and prevented.

Conventional methods for the identification of AMRs involve microbial isolation (via culture) and phenotypic typing, which together can take a few days or weeks to complete [41]. Moreover, not all microbial species are amenable to laboratory-based culturing [42]. DNA-sequencing technologies may be used to sequence the genomes of cultured micro-organisms for the presence of AMR genes, which reduces the time-to-result time. Currently, Illumina sequencing is most widely used, but using this sequencing technology generates difficulties in correctly identifying repetitive insertion sequences, sequences that may flank horizontally acquired genes associated with AMR [43]. Nanopore long-read assemblies could improve resolving these repetitive regions.

#### Use case 1: Long-read sequencing analysis

The NanoGalaxy toolkit incorporates a rapid long-read assembly workflow using minimap2 [22], miniasm [18], and Racon [44]. Tools for further analysis in the toolkit include Staramr [35] for resistance gene detection, PlasFlow [34] and Bandage [31] for microbial species/plasmid determination, and NanoPlot [30] for quality assessment.

In this worked example, the outcome of the NanoGalaxy pipeline was compared to the plasmid sequences recovered by Li et al. [45] (Supplementary Table S1). The pipeline recovered 19 of 21 plasmids, with a mean identity of 97.76%. The number of detected resistance genes was higher than that found by Li et al. [45], which was expected because Staramr [35] includes the PointFinder (chromosomal point mutations) database [46] and current long-read sequencing may generate relatively high sequence error rates.

#### Use case 2: Combining short- and long-read sequencing

The previously described long-read assembly workflow rapidly assembles genomes. Because short-read sequencing platforms tend to have a higher accuracy at single-nucleotide level, hybrid solutions to gain from both short- and long-read data are of special interest. The NanoGalaxy toolkit includes a workflow that processes both long- and short-read sequences. In this respect, Unicycler was integrated into the NanoGalaxy toolkit to combine the best features of long- and short-read sequencing technologies. The workflow recommended by the Unicycler developers [16] includes Trim Galore [47], Porechop [26], and Filtlong [27] for quality trimming; Unicycler [16] for*de novo* assembly; and Bandage [31] for plasmid visualization. These tools are available as stand-alone tools and combined in a NanoGalaxy workflow.

**Figure 1:**
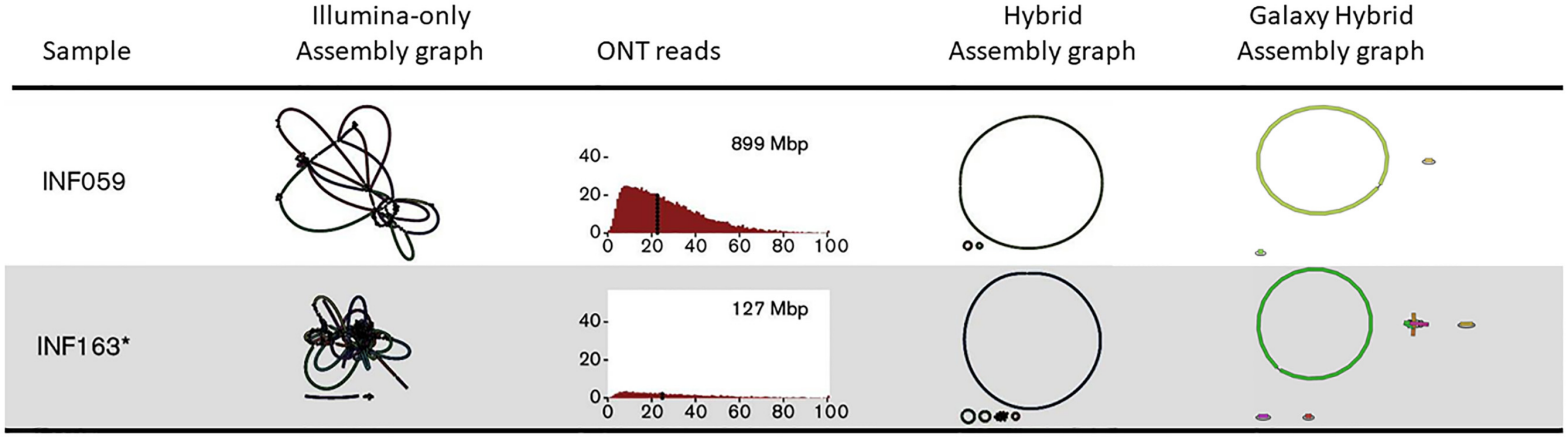
Representation of the output of Wick et al. [16]. The plasmid assembly graphs output created by Bandage [31] are shown to confirm that the workflow functions as expected. The length distribution, total yield, and N50 of the Oxford Nanopore Technologies (ONT) reads of each *Klebsiella pneumoniae* represent the input data. Mb: megabase pairs.

The assembly graphs shown in Fig. 1 compare the NanoGalaxy toolkit with the results from Wick et al. [16]. The Illumina-only (short-read sequencing) graphs show no clear structure(s) present, whereas Nanopore-only (long-read sequencing) is able to generate the circularized structure expected of plasmids. The combination of both sequence techniques gives the clearest view of the circular assemblage expected of plasmids, analogues to the results obtained by Wick et al. [16] (Fig. 1). Note that different combinations of short- and long-read tools can be used individually or combined to generate personalized workflows.

## Conclusion

In this work we covered some important aspects of long-read sequencing analysis with a special focus on ONT sequencing data. We aggregated commonly used tools into a single consistent interoperable interface and presented solutions for metagenomic analysis and genome assembly. Furthermore, other long-read sequencing data analysis tools have been developed or are currently under development; however, we have focused on the most established and widely used tools. Nevertheless, we expect that the toolkit will be further extended by the community because NanoGalaxy is part of the open Galaxy platform and Galaxy community. Last, the majority of the integrated tools that support other technologies such as Pacific Biosciences should also work inside Galaxy. However, here we have performed intensive testing of the integrated tools for ONT data.

## Methods

### Implementation

The tools and workflows included in the NanoGalaxy toolkit enable non-bioinformatics-trained researchers to perform extensive genomics analysis using long-read sequence data, without the need for any coding skills. All tools and their dependencies are installed on the Galaxy platform and are managed by the Conda framework for dependency management. NanoGalaxy tools and their dependencies are available from the Bioconda Conda channel [48]. The Galaxy wrappers are developed openly on GitHub, utilizing the Travis continuous integration framework [49] for testing, and have been made available on the Galaxy ToolShed [13].

### Training Materials

An online training manual for the AMR use case described in this publication, as well as a description of NanoGalaxy tools and end-to-end workflows, can be found on the Galaxy training materials website [50].

### Future Work

The availability of long-read sequencing platforms and data analysis tools is relatively new, with improvements in technology and software continually being developed. As more tools become available these will need to be assembled into existing or new toolkits. Additionally, the future availability of toolkits such as NanoGalaxy will help popularize long-read sequencing, while making it accessible to non-bioinformatics-trained researchers of the future.

## Availability of Source Code and Requirements

- Project name: NanoGalaxy
- Project home page: https://nanopore.usegalaxy.eu
- Training Manual: https://training.galaxyproject.org/training-material/topics/metagenomics/tutorials/plasmid-metagenomics-nanopore/tutorial.html
- License: GNU GPL
- BiotoolsID: nanogalaxy
- RRID: SCR_018912

All developed Galaxy wrappers are available for installation from the Galaxy Tool Shed (https://toolshed.g2.bx.psu.edu/). The corresponding code repositories for the tool wrappers are listed in Table 2. The workflows described in this work are publicly available from the European Galaxy server, as well as published Galaxy histories with an example run of each of these workflows (Table 3).

**Table 2:** Tool availability

Tool	Github repository
Bandage	https://github.com/galaxyproject/tools-iuc/tree/master/tools/bandage
Canu	https://github.com/bgruening/galaxytools/tree/master/tools/canu
Circos	https://github.com/galaxyproject/tools-iuc/tree/master/tools/circos
Filtlong	https://github.com/galaxyproject/tools-iuc/tree/master/tools/filtlong
Flye	https://github.com/bgruening/galaxytools/tree/master/tools/flye
GraphMap	https://github.com/bgruening/galaxytools/tree/master/tools/graphmap
Kraken2	https://github.com/galaxyproject/tools-iuc/tree/master/tool_collections/kraken2/kraken2
Medaka	https://github.com/galaxyproject/tools-iuc/tree/master/tools/medaka
Miniasm	https://github.com/galaxyproject/tools-iuc/tree/master/tools/miniasm
Minimap2	https://github.com/galaxyproject/tools-iuc/tree/master/tools/minimap2
Nanoplot	https://github.com/galaxyproject/tools-iuc/tree/master/tools/nanoplot
Nanopolish	https://github.com/bgruening/galaxytools/tree/master/tools/nanopolish
NanopolishComp	https://github.com/galaxyproject/tools-iuc/tree/master/tools/nanopolishcomp
Ont_fast5_api	https://github.com/galaxyproject/tools-iuc/tree/master/tools/ont_fast5_api
Pilon	https://github.com/galaxyproject/tools-iuc/tree/master/tools/pilon
PlasFlow	https://github.com/galaxyproject/tools-iuc/tree/master/tools/plasflow
Porechop	https://github.com/galaxyproject/tools-iuc/tree/master/tools/porechop
Poretools	https://github.com/galaxyproject/tools-iuc/tree/master/tools/poretools
Unicycler	https://github.com/galaxyproject/tools-iuc/tree/master/tools/unicycler
Racon	https://github.com/bgruening/galaxytools/tree/master/tools/racon
Spades	https://github.com/galaxyproject/tools-iuc/tree/master/tools/spades
Staramr	https://github.com/phac-nml/galaxy_tools/tree/master/tools/staramr
Wtdbg2	https://github.com/bgruening/galaxytools/tree/master/tools/wtdbg

**Table 3:** Workflow availability

Workflow	Link	History	SEEK ID
Basic workflows inspired by the Nanopolish tutorials	https://nanopore.usegalaxy.eu/u/milad/w/nanopolish-variants-tutorial	https://usegalaxy.eu/u/milad/h/nanopolish-tutorial	https://workflowhub.eu/workflows/50?version=1
Genome assembly: Flye-based WF for highly repetitive genomes [37]	https://nanopore.usegalaxy.eu/u/milad/w/ont-assembly-flye-ahrens	https://usegalaxy.eu/u/milad/h/ahrens-nanopore-gmmap	https://workflowhub.eu/workflows/51?version=1
Genome assembly: Unicycler-based WF for *Klebsiella pneumoniae* [51]	https://usegalaxy.eu/u/milad/h/wick-etal-nanopore	https://usegalaxy.eu/u/milad/h/wick-etal-nanopore	https://workflowhub.eu/workflows/52?version=1
Metagenomics: taxa classification	https://nanopore.usegalaxy.eu/u/milad/w/metagenomics-krakan2	https://usegalaxy.eu/u/milad/h/nanoporebeerdecodechimaytriple	https://workflowhub.eu/workflows/53?version=1

WF: workflow.

### Galaxy Resources

- Galaxy Home Page: https://galaxyproject.org/
- Galaxy Tutorials: https://training.galaxyproject.org
- How to install Galaxy: https://getgalaxy.org
- How to install tools: https://galaxyproject.org/admin/tools/add-tool-from-toolshed-tutorial/
- Full administrative resources: https://docs.galaxyproject.org/
- Galaxy Help Forum: https://help.galaxyproject.org/
- Connect with the Galaxy Community on Gitter Chat: https://gitter.im/galaxyproject/Lobby/

## Availability of Supporting Data and Materials

The data presented here to illustrate the functionality of the tools were obtained from previous publications [45,51] and were collected and made available from Zenodo [52].

Additional supporting data are available from the *GigaScience* GigaDB database [53].

## Additional Files

Supplementary Table S1. The plasmids found by the workflow are BLAST against the plasmid recovered by R. Li et al.

## Abbreviations

AMR: antimicrobial resistance; bp: base pairs; kb: kilobase pairs; ONT: Oxford Nanopore Technologies; SNP: single-nucleotide polymorphism.

## Competing Interests

The authors declare that they have no competing interests.

## Funding

This project was made possible with the support of Support Casper and the Albert Ludwig University of Freiburg. This project has received funding from the European Union's Horizon 2020 research and innovation programme under grant agreement 825775.

## Authors' Contributions

W.d.K., M.M., and S.H. contributed to toolkit development and writing of the manuscript. A.H. tested and evaluated the tools and suggested modifications, feature requests, and user improvements. J.P.H. contributed to AMR tool and nanopore sequencing discussions and the writing of the manuscript. M.v.d.B. and S.F. contributed to the tool development. B.G. contributed to the tool development and manuscript writing and supervised the project. D.A.M., R.B., and A.P.S. supervised the project. All authors approved the final version of the manuscript.

## Supplementary Material

giaa105_GIGA-D-20-00112_Original_Submission

giaa105_GIGA-D-20-00112_Revision_1

giaa105_GIGA-D-20-00112_Revision_2

giaa105_Response_to_Reviewer_Comments_Original_Submission

giaa105_Response_to_Reviewer_Comments_Revision_1

giaa105_Reviewer_1_Report_Original_Submission Wouter De Coster -- 4/24/2020 Reviewed

giaa105_Reviewer_2_Report_Original_Submission David Eccles -- 5/10/2020 Reviewed

giaa105_Reviewer_2_Report_Revision_1 David Eccles -- 8/30/2020 Reviewed

giaa105_Reviewer_3_Report_Original_Submission Federico Zambelli -- 5/12/2020 Reviewed

giaa105_Reviewer_3_Report_Revision_1 Federico Zambelli -- 8/19/2020 Reviewed

giaa105_Supplemental_Files

## References

[1] Gilissen C , Hoischen A, Brunner HG, et al. Unlocking Mendelian disease using exome sequencing. Genome Biol. 2011;12(9):228. 21920049 10.1186/gb-2011-12-9-228 PMC3308044

[2] de Koning AJ , Gu W, Castoe TA, et al. Repetitive elements may comprise over two-thirds of the human genome. PLoS Genet. 2011;7(12):e1002384. 22144907 10.1371/journal.pgen.1002384 PMC3228813

[3] Goodwin S , McPherson JD, McCombie WR. Coming of age: Ten years of next-generation sequencing technologies. Nat Rev Genet. 2016;17(6):333. 27184599 10.1038/nrg.2016.49 PMC10373632

[4] Feuk L , Carson AR, Scherer SW. Structural variation in the human genome. Nat Rev Genet. 2006;7(2):85. 16418744 10.1038/nrg1767

[5] Jain M , Olsen HE, Paten B, et al. The Oxford Nanopore MinION: Delivery of nanopore sequencing to the genomics community. Genome Biol. 2016;17(1):239. 27887629 10.1186/s13059-016-1103-0 PMC5124260

[6] Rhoads A , Au KF. PacBio sequencing and its applications. Genomics Proteomics Bioinformatics. 2015;13(5):278–89. 26542840 10.1016/j.gpb.2015.08.002 PMC4678779

[7] Tsai YC , Greenberg D, Powell J, et al. Amplification-free, CRISPR-Cas9 targeted enrichment and SMRT sequencing of repeat-expansion disease causative genomic regions. bioRxiv. 2017:203919.

[8] Flusberg BA , Webster DR, Lee JH, et al. Direct detection of DNA methylation during single-molecule, real-time sequencing. Nat Methods. 2010;7(6):461. 20453866 10.1038/nmeth.1459 PMC2879396

[9] Köster J , Rahmann S. Snakemake—A scalable bioinformatics workflow engine. Bioinformatics. 2012;28(19):2520–2. 22908215 10.1093/bioinformatics/bts480

[10] Di Tommaso P , Chatzou M, Floden EW, et al. Nextflow enables reproducible computational workflows. Nat Biotechnol. 2017;35(4):316–9. 28398311 10.1038/nbt.3820

[11] Afgan E , Baker D, Batut B, et al. The Galaxy platform for accessible, reproducible and collaborative biomedical analyses: 2018 update. Nucleic Acids Res. 2018;46(W1):W537–44. 29790989 10.1093/nar/gky379 PMC6030816

[12] Zotero: Galaxy. https://www.zotero.org/groups/1732893/galaxy. Accessed: 20-06-2019.

[13] Galaxy Tool Shed. https://toolshed.g2.bx.psu.edu/. Accessed: 20-06-2019.

[14] Kolmogorov M , Yuan J, Lin Y, et al. Assembly of long, error-prone reads using repeat graphs. Nat Biotechnol. 2019;37(5):540. 30936562 10.1038/s41587-019-0072-8

[15] Koren S , Walenz BP, Berlin K, et al. Canu: Scalable and accurate long-read assembly via adaptive k-mer weighting and repeat separation. Genome Res. 2017;27(5):722–36. 28298431 10.1101/gr.215087.116 PMC5411767

[16] Wick RR , Judd LM, Gorrie CL, et al. Unicycler: Resolving bacterial genome assemblies from short and long sequencing reads. PLoS Comput Biol. 2017;13(6):e1005595. 28594827 10.1371/journal.pcbi.1005595 PMC5481147

[17] Ruan J , Li H. Fast and accurate long-read assembly with wtdbg2. Nat Methods. 2020;17:155–8. 31819265 10.1038/s41592-019-0669-3 PMC7004874

[18] Li H . Minimap and miniasm: Fast mapping and de novo assembly for noisy long sequences. Bioinformatics. 2016;32(14):2103–10. 27153593 10.1093/bioinformatics/btw152 PMC4937194

[19] Vaser R , Sović I, Nagarajan N, et al. Fast and accurate de novo genome assembly from long uncorrected reads. Genome Res. 2017;27(5):737–46. 28100585 10.1101/gr.214270.116 PMC5411768

[20] Nurk S , Bankevich A, Antipov D, et al. Assembling genomes and mini-metagenomes from highly chimeric reads. In: Annual International Conference on Research in Computational Molecular Biology. Springer; 2013:158–70.

[21] Oxford Nanopore Technologies. Medaka. GitHub. 2018. https://github.com/nanoporetech/medaka.

[22] Li H . Minimap2: Pairwise alignment for nucleotide sequences. Bioinformatics. 2018;34(18):3094–100. 29750242 10.1093/bioinformatics/bty191 PMC6137996

[23] Sović I , Šikić M, Wilm A, et al. Fast and sensitive mapping of nanopore sequencing reads with GraphMap. Nat Commun. 2016;7:11307. 27079541 10.1038/ncomms11307 PMC4835549

[24] Oxford Nanopore Technologies. ont_fast5_api. GitHub. 2019. https://github.com/nanoporetech/ont_fast5_api.

[25] Loman NJ , Quick J, Simpson JT. A complete bacterial genome assembled de novo using only nanopore sequencing data. Nat Methods. 2015;12(8):733. 26076426 10.1038/nmeth.3444

[26] Wick R . Porechop. Github. 2017. https://github.com/rrwick/Porechop.

[27] Wick R . Filtlong. Github. 2017. https://github.com/rrwick/Filtlong.

[28] Loman NJ , Quinlan AR. Poretools: A toolkit for analyzing nanopore sequence data. Bioinformatics. 2014;30(23):3399–401. 25143291 10.1093/bioinformatics/btu555 PMC4296151

[29] Walker BJ , Abeel T, Shea T, et al. Pilon: An integrated tool for comprehensive microbial variant detection and genome assembly improvement. PLoS One. 2014;9(11):e112963. 25409509 10.1371/journal.pone.0112963 PMC4237348

[30] De Coster W , D'Hert S, Schultz DT, et al. NanoPack: Visualizing and processing long-read sequencing data. Bioinformatics. 2018;34(15):2666–9. 29547981 10.1093/bioinformatics/bty149 PMC6061794

[31] Wick RR , Schultz MB, Zobel J, et al. Bandage: Interactive visualization of de novo genome assemblies. Bioinformatics. 2015;31(20):3350–2. 26099265 10.1093/bioinformatics/btv383 PMC4595904

[32] Krzywinski MI , Schein JE, Birol I, et al. Circos: An information aesthetic for comparative genomics. Genome Res. 2009;19:1639–45. 19541911 10.1101/gr.092759.109 PMC2752132

[33] Wood DE , Lu J, Langmead B. Improved metagenomic analysis with Kraken 2. Genome Biol. 2019;20:257. 31779668 10.1186/s13059-019-1891-0 PMC6883579

[34] Krawczyk PS , Lipinski L, Dziembowski A. PlasFlow: Predicting plasmid sequences in metagenomic data using genome signatures. Nucleic Acids Res. 2018;46(6):e35. 29346586 10.1093/nar/gkx1321 PMC5887522

[35] Staramr. GitHub. 2018. https://github.com/phac-nml/staramr.

[36] Ondov BD , Bergman NH, Phillippy AM. Interactive metagenomic visualization in a Web browser. BMC Bioinformatics. 2011;12(1):385. 21961884 10.1186/1471-2105-12-385 PMC3190407

[37] Schmid M , Frei D, Patrignani A, et al. Pushing the limits of de novo genome assembly for complex prokaryotic genomes harboring very long, near identical repeats. Nucleic Acids Res. 2018;46(17):8953–65. 30137508 10.1093/nar/gky726 PMC6158609

[38] Organisation for Economic Co-operation and Development , https://www.oecd.org/health/health-systems/AMR-Policy-Insights-November2016.pdf. Accessed: 23-06-2019. Antimicrobial Resistance. 2017.

[39] World Health Organization. Antibiotic resistance, https://www.who.int/news-room/fact-sheets/detail/antibiotic-resistance. Accessed: 23-06-2019. 2018.

[40] O'Neill J . Antimicrobial resistance: Tackling a crisis for the health and wealth of nations. Review on Antimicrobial Resistance. London, UK: Review on Antimicrobial Resistance; 2014. https://amr-review.org/Publications.html.

[41] Quick J , Ashton P, Calus S, et al. Rapid draft sequencing and real-time nanopore sequencing in a hospital outbreak of Salmonella. Genome Biol. 2015;16(1):114. 26025440 10.1186/s13059-015-0677-2 PMC4702336

[42] Mitsuhashi S , Kryukov K, Nakagawa S, et al. A portable system for rapid bacterial composition analysis using a nanopore-based sequencer and laptop computer. Sci Rep. 2017;7:5657. 28720805 10.1038/s41598-017-05772-5 PMC5516037

[43] Ashton PM , Nair S, Dallman T, et al. MinION nanopore sequencing identifies the position and structure of a bacterial antibiotic resistance island. Nat Biotechnol. 2014;33:296. 25485618 10.1038/nbt.3103

[44] Vaser R , Sović I, Nagarajan N, et al. Fast and accurate de novo genome assembly from long uncorrected reads. Genome Res. 2017;27(5):737–46. 28100585 10.1101/gr.214270.116 PMC5411768

[45] Li R , Xie M, Dong N, et al. Efficient generation of complete sequences of MDR-encoding plasmids by rapid assembly of MinION barcoding sequencing data. Gigascience. 2018;7(3), doi:10.1093/gigascience/gix132. PMC5848804 29325009

[46] Zankari E , Allesøe R, Joensen KG, et al. PointFinder: A novel web tool for WGS-based detection of antimicrobial resistance associated with chromosomal point mutations in bacterial pathogens. J Antimicrob Chemother. 2017;72(10):2764–8. 29091202 10.1093/jac/dkx217 PMC5890747

[47] Kreuger F . Trim Galore! Github. 2016. https://github.com/FelixKrueger/TrimGalore.

[48] Grüning B , Dale R, Sjödin A, et al. Bioconda: Sustainable and comprehensive software distribution for the life sciences. Nat Methods. 2018;15(7):475. 29967506 10.1038/s41592-018-0046-7 PMC11070151

[49] Travis CI: Test and Deploy with Confidence. https://travis-ci.org/ . Accessed 1st July 2020

[50] Batut B , Hiltemann S, Bagnacani A, et al. Community-driven data analysis training for biology. Cell Syst. 2018;6(6):752–8. 29953864 10.1016/j.cels.2018.05.012 PMC6296361

[51] Wick RR , Judd LM, Gorrie CL, et al. Completing bacterial genome assemblies with multiplex MinION sequencing. Microb Genom. 2017;3(10):e000132. 29177090 10.1099/mgen.0.000132 PMC5695209

[52] de Koning W , Miladi M, et al. Zenodo: “NanoGalaxy: Nanopore long-read sequencing data analysis in Galaxy". Zenodo. 2020. 10.5281/zenodo.3741446. PMC7568507 33068114

[53] de Koning W , Miladi M, Hiltemann S, et al. Supporting data for “NanoGalaxy: Nanopore long-read sequencing data analysis in Galaxy.”. GigaScience Database. 2020. 10.5524/100795. PMC7568507 33068114

