# Supplementary material for: NanoGalaxy: Nanopore long-read sequencing data analysis in Galaxy
Source: Gigascience. 2020 Oct 17;9(10):giaa105. doi: 10.1093/gigascience/giaa105 (PMC7568507; doi:10.1093/gigascience/giaa105)
Supplement: giaa105_GIGA-D-20-00112_Revision_2 [file giaa105_giga-d-20-00112_revision_2.pdf]

GigaScience

NanoGalaxy: Nanopore long-read sequencing data analysis in Galaxy

--Manuscript Draft--

**Manuscript Number:**	GIGA-D-20-00112R2
**Full Title:**	NanoGalaxy: Nanopore long-read sequencing data analysis in Galaxy
**Article Type:**	Technical Note
**Funding Information:**	Horizon 2020 research and innovation programme (825775) — Mr. Willem de Koning; Ms. Saskia Hiltemann; Dr. Andrew P. Stubbs
**Abstract:**	Background: Long-read sequencing can be applied to generate very long contigs and even completely assembled genomes at relatively low cost and with minimal sample preparation. As a result, long-read sequencing platforms are becoming more popular. In this respect, the Oxford Nanopore Technologies-based long-read sequencing 'nanopore' platform is becoming a widely used tool with a broad range of applications and end-users. However, the need to explore and manipulate the specialised data generated by long-read sequencing platforms means that accompanying specialised bioinformatics platforms and tools are required in order to correctly process the long-read data generated. Importantly, such tools should additionally help democratise bioinformatics analysis by enabling easy access and ease-of-use solutions for researchers.<br>Results: The Galaxy platform provides a user-friendly interface to computational command-line-based tools, including their software dependencies and refined workflows. The interface enables researchers, who do not necessarily possess programming experience or extended computer skills, to perform powerful bioinformatics analysis, including the assembly and analysis of short- or long-read sequence data. The newly developed 'NanoGalaxy' is a Galaxy-based toolkit for analysing long-read sequencing data, which is suitable for diverse applications, including de novo genome assembly from genomic, metagenomic and plasmid sequence reads.<br>Conclusions: A range of best practice tools and workflows for long-read sequence genome assembly have been integrated into a NanoGalaxy platform in order to facilitate easy access and use of bioinformatics tools for researchers. Nanogalaxy is freely available at the European Galaxy server nanopore.usegalaxy.eu with supporting self-learning training material available at training.galaxyproject.org .
**Corresponding Author:**	Willem de Koning, M.Sc.<br>Erasmus MC<br>Rotterdam, NETHERLANDS
**Corresponding Author Secondary Information:**	
**Corresponding Author's Institution:**	Erasmus MC
**Corresponding Author's Secondary Institution:**	
**First Author:**	Willem de Koning, M.Sc.
**First Author Secondary Information:**	
**Order of Authors:**	Willem de Koning, M.Sc.
	Milad Miladi
	Saskia Hiltemann
	Astrid Heikema
	John P. Hays
	Stephan Flemming
	Marius van den Beek
	Dana A. Mustafa

Powered by Editorial Manager® and ProduXion Manager® from Aries Systems Corporation

	Rolf Backofen
	Björn Grüning
	Andrew P. Stubbs
**Order of Authors Secondary Information:**	

Response to Reviewers:

Dear Scott,

I have added the DOI and reference in the availability of supporting data section.

All the best,
Willem

Dear Scott,

I would like to thank you for considering our manuscript “NanoGalaxy: Nanopore long-read sequencing data analysis in Galaxy”. I have changed the manuscript according to the minor comments of David.

Furthermore, I sent an email to the curator with the information he asked for.
Hopefully this is sufficient to finalize the manuscript. Otherwise, please let me know.

All the best,
Willem

Dear David,

Thanks for your new review regarding our manuscript. Below you’ll find a point-by-point description of the changes made according to your new comments and suggestions.

Overall summary: I'm happy that most of the issues I raised about the manuscript have been dealt with, and am okay with recommending publication as-is, given that additional issues I have are relatively minor. While there are issues with some tools, that is expected from an initial release, and I expect that a suitably inquisitive reader will still be able to get their feet wet using NanoGalaxy. Thank you for giving me an opportunity to explore NanoGalaxy.

I’m glad you appreciate the opportunity to explore NanoGalaxy and want to thank you for recommending the publication. Nevertheless, we changed the manuscript according to your minor comments.

As far as I can tell, the primary publication for Galaxy (Afgan et al., 2018) is not directly cited, nor is the original 2005 paper cited (although on reflection, the 2018 paper would be a better citation). It would be a good idea to include a citation specific to Galaxy, as recommended in the NanoGalaxy 'How to Cite Galaxy' information:
https://eur01.safelinks.protection.outlook.com/?url=https%3A%2F%2Fgalaxyproject.org%2Fciting-galaxy%2F%23primary-publication&data=02%7C01%7Cw.dekoning.1%40erasmusmc.nl%7Cb1868667c5924c1ef0f108d84d7320cd%7C526638ba6af34b0fa532a1a511f4ac80%7C0%7C0%7C637344504151659246&sdata=5K2q81Ool9f%2FlSDqh1ERnd2TYwAEJbq3QHd%2F7ektPz8%3D&reserved=0 The Galaxy citation would be best in the first sentence of the last paragraph of the introduction. According to that page, the citation for Galaxy Toolshed [reference #12 in the manuscript] should be Blankenberg et al., 2014.

Thanks again for pointing out this issue, and expanding the explanation. Indeed, we added the citation on the suggested location in the manuscript as it is appropriate.

-- Software / Pipeline evaluation --
Now that I can load datasets in NanoGalaxy, I had another go at using NanoGalaxy blindly with other datasets:
1) used 'fasterq-dump' to retrieve reads from SRR12395855

2) Changed dataset type to 'fastq.gz'
3) Ran 'NanoPlot' on dataset collection 'Single-end data (fasterq-dump)'
4) Run 'Flye Assembly' with estimated genome size of 5m [tool error "cannot find 'ext'"]
5) Run 'Canu assembler' with estimated genome size of 5m
[Stopped after waiting ~16h for job completion]
[I didn't run miniasm, because I didn't know at this stage how to do an all-vs-all mapping via NanoGalaxy]
1) used 'fasterq-dump' to retrieve reads from SRR4734731
[error: "accession 'SRR4734731' is PACBIO, please use fastq-dump instead"]
2) used 'fastq-dump' to retrieve reads from SRR4734731
3) Run 'Canu assembler' with an estimated genome size of 16k.
[Tried to set stopOnLowCoverage=0, but this option wouldn't be accepted]
On further reflection, it would help if assembly tools would give a warning (especially for datasets > 100 Mb) about the time taken for the assembly step.

Thank you for trying our tools, we have added the following note to the assembly tools based on your feedback: "NOTE: This tool may take a long time depending on the size and characteristics of your dataset."

Given troubles with my own datasets, I tried to find / test out the "Use Case 1: Long Read Sequencing Analysis" workflow. Unfortunately, the manuscript doesn't include information about input datasets. It mentions "In this worked example", but I can't see a worked example anywhere. Earlier in the manuscript, I see a reference to the Galaxy training materials website, https://eur01.safelinks.protection.outlook.com/?url=https%3A%2F%2Ftraining.galaxyproject.org%2F&data=02%7C01%7Cw.dekoning.1%40erasmusmc.nl%7Cb1868667c5924c1ef0f108d84d7320cd%7C526638ba6af34b0fa532a1a511f4ac80%7C0%7C0%7C637344504151659246&sdata=LD8F60MoLA4UgJdQ39CZnXnwEYeN054Oh4cdckEtjsY%3D&reserved=0, and eventually found (after searching for "nanopore") a Galaxy Training workflow with the title "Antibiotic resistance detection", with authors matching the authors of this manuscript. That title doesn't seem to be anywhere in the manuscript.
It would be helpful if a direct link to this workflow were added to the paper, or at the very least a reference to the workflow title, "Antibiotic resistance detection":
https://eur01.safelinks.protection.outlook.com/?url=https%3A%2F%2Ftraining.galaxyproject.org%2Ftraining-material%2Ftopics%2Fmetagenomics%2Ftutorials%2Fplasmid-metagenomics-nanopore%2Ftutorial.html&data=02%7C01%7Cw.dekoning.1%40erasmusmc.nl%7Cb1868667c5924c1ef0f108d84d7320cd%7C526638ba6af34b0fa532a1a511f4ac80%7C0%7C0%7C637344504151669239&sdata=81iS4K%2FwWRI31RcWL%2BYIRLszQLRNIoBrudpY%2BNybcb0%3D&reserved=0

Indeed the Galaxy training website is mentioned, but not the name of the related training. Therefore, the name of the training is added to the manuscript.

After accessing and reading this workflow, I was able to run through the example successfully and carry out all operations:
* running nanoplot
* running all-vs-all minimap2 on the collected dataset
* generating an assembly with miniasm
* converting the resultant GFA to FASTA format
* remapping reads to the assembled genomes with minimap2
* consensus calling with Racon
* visualising miniasm assembly graph with Bandage
* Assembling using unicycler with nanopore-only reads
Unfortunately, I couldn't find 'PlasFlow' or 'staramr' in the Tools search to attempt those steps. It would be a good idea to either add these to NanoGalaxy (in a way that they're visible by search), or create an alternative workflow that stops at the assembly step.

The metagenomics analysis tools indeed seemed to be missing on the website, therefore this category is added to nanopore.usegalaxy.eu.

Summary: NanoGalaxy is at least loading input datasets, and some tools work. Importantly, the tools in the specified workflow are working for generating a full

Powered by Editorial Manager® and ProduXion Manager® from Aries Systems Corporation

	assembly, when using the parameters stated in the workflow. I expect that the inconsistency with toolsets will improve over time as more tool paths get more eyes and more use.<br><br>We hope that our changes improve the manuscript as expected.<br><br>All the best,<br>Willem
Additional Information:	
Question	Response
Are you submitting this manuscript to a special series or article collection?	No
**Experimental design and statistics**<br><br>Full details of the experimental design and statistical methods used should be given in the Methods section, as detailed in our Minimum Standards Reporting Checklist. Information essential to interpreting the data presented should be made available in the figure legends.<br><br>Have you included all the information requested in your manuscript?	Yes
**Resources**<br><br>A description of all resources used, including antibodies, cell lines, animals and software tools, with enough information to allow them to be uniquely identified, should be included in the Methods section. Authors are strongly encouraged to cite Research Resource Identifiers (RRIDs) for antibodies, model organisms and tools, where possible.<br><br>Have you included the information requested as detailed in our Minimum Standards Reporting Checklist?	Yes
**Availability of data and materials**<br><br>All datasets and code on which the conclusions of the paper rely must be either included in your submission or	Yes

deposited in publicly available repositories (where available and ethically appropriate), referencing such data using a unique identifier in the references and in the "Availability of Data and Materials" section of your manuscript.<br><br>Have you have met the above requirement as detailed in our Minimum Standards Reporting Checklist?	

Powered by Editorial Manager® and ProduXion Manager® from Aries Systems Corporation

Click here to access/download;Manuscript;main.tex

GigaScience, 2017, 1–7

doi: xx.xxxx/xxxx
Manuscript in Preparation
Technical Note

TECHNICAL NOTE

NanoGalaxy: Nanopore long-read sequencing data analysis in Galaxy

Willem de Koning[1,2,†*], Milad Miladi[3,†], Saskia Hiltemann[1], Astrid Heikema[4], John P. Hays[4], Stephan Flemming[3], Marius van den Beek[5], Dana A. Mustafa[2], Rolf Backofen[3], Björn Grüning[3,*] and Andrew P. Stubbs[1]

[1]Department of Pathology, Clinical Bioinformatics Unit, Erasmus University Medical Centre, Wytemaweg 80, 3015 CN, Rotterdam, The Netherlands and [2]Department of Pathology, Tumor Immuno-Pathology Laboratory, Erasmus University Medical Centre, ‘s Gravendijkwal 230, 3015 CE, Rotterdam, The Netherlands and [3]Department of Computer Science, Bioinformatics Group, University of Freiburg, 79110 Freiburg im Breisgau, Germany and [4]Department of Medical Microbiology and Infectious Diseases, Erasmus University Medical Centre, ‘s Gravendijkwal 230, 3015 CE, Rotterdam, The Netherlands and [5]Department of Stem Cells and Tissue Homeostasis, Institut Curie, PSL Research University, 75005 Paris, France

[†]Contributed equally.
[*] To whom correspondence should be addressed. w.dekoning.1@erasmusmc.nl; gruening@informatik.uni-freiburg.de

Abstract

Background: Long-read sequencing can be applied to generate very long contigs and even completely assembled genomes at relatively low cost and with minimal sample preparation. As a result, long-read sequencing platforms are becoming more popular. In this respect, the Oxford Nanopore Technologies-based long-read sequencing ’nanopore’ platform is becoming a widely used tool with a broad range of applications and end-users. However, the need to explore and manipulate the complex data generated by long-read sequencing platforms requires accompanying specialised bioinformatics platforms and tools in order to process the long-read data correctly. Importantly, such tools should additionally help democratise bioinformatics analysis by enabling easy access and ease-of-use solutions for researchers.
Results: The Galaxy platform provides a user-friendly interface to computational command-line-based tools, handles the software dependencies and provides refined workflows. The users do not necessarily have to possess programming experience or extended computer skills. The interface enables researchers to perform powerful bioinformatics analysis, including the assembly and analysis of short- or long-read sequence data. The newly developed ‘NanoGalaxy’ is a Galaxy-based toolkit for analysing long-read sequencing data, which is suitable for diverse applications, including de novo genome assembly from genomic, metagenomic and plasmid sequence reads.
Conclusions: A range of best practice tools and workflows for long-read sequence genome assembly has been integrated into a NanoGalaxy platform in order to facilitate easy access and use of bioinformatics tools for researchers. Nanogalaxy is freely available at the European Galaxy server `https://nanopore.usegalaxy.eu` with supporting self-learning training material available at `https://training.galaxyproject.org`.

Key words: Long-read sequencing; Nanopore; Galaxy; Reproducibility; Workflows

Compiled on: September 15, 2020.
Draft manuscript prepared by the author.

Background

Short-read sequencing has become a routine technique within clinical diagnostics [1]. However, the short length of the reads obtained (150-300 bp) complicates the assembly of genomes, especially for highly repetitive regions and the detection of structural variation [2, 3, 4]. Furthermore, even "state-of-the-art" algorithms cannot overcome the issues associated with genome mapping or assembly using short-read sequences. Importantly, advances in sequencing technology now allow *long-read sequencing* to be performed. The two prominent long-read sequencing platforms are nanopore sequencing by Oxford Nanopore Technologies and single molecule real time sequencing by PacBio [5, 6]. These platforms generate sequence reads much longer than the classic short-read technologies, including long-reads from single DNA molecules and without the need of PCR amplification (>10 kilobase on average). Moreover, utilising these technologies, library preparation and sequencing may be performed outside of traditional research laboratories, with sequencing outputs generated in real-time [7]. Protocols that require no PCR amplification also permit the direct detection of base modifications [8].

Analyzing the large amount of data generated by the short- and long-read sequencing technologies is a complex, multi-step process that is computationally intensive and often require bioinformatics expertise. Specifically, for each step in the analysis, a set of different tools or software may be needed. For example, *de novo* assembly is performed via a combination of multiple alignments, assembly and polishing tools, each utilizing its own input parameters. Such tools are typically executed from a UNIX command-line and require extensive computational resources, adding to the complexity of the analysis process. Command-line based workflow managers such as Snakemake and Nextflow [9, 10] can be used for analysing the data. However, these solutions require having expertise in working from the command line. On the other side, some web-based solutions have also been offered. For example EPI2ME platform offers a cloud-based solution with a web interface. The platform supports practical solutions for a limited set of application scenarios, and provides a limited flexibility for configuring the underlying workflows. Here, the Galaxy platforms offers a flexible data analysis platform with a high degree of flexibility, similar to the command-line based workflow managers, and an accessible web interface.

The Galaxy platform reduces the data analysis complexity and implements a standardized and user-friendly interface that accommodates command-line tools and refined workflows complete with their dependencies [11]. The platform hosts a wide range of tools/software and is widely used for bioinformatics analysis within the biological science community [12, 13]. Here we introduce the NanoGalaxy toolkit for analysing Nanopore long-read data. NanoGalaxy comprises a series of integrated Galaxy-based tools that enable researchers to generate powerful short- or long-sequence read assemblies for genomic and plasmid bioinformatics analyses. The NanoGalaxy toolkit is a user-friendly environment that can be utilized inside or outside of traditional research laboratories.

Findings

Tools

We have integrated a large collection of long-read sequence tools into the Galaxy platform, the NanoGalaxy toolkit, including diverse applications for the analyses of long-read sequences (Table 1). This toolkit is freely available from the Galaxy ToolShed, and has additionally been made available as a specialized GalaxyEU subdomain (https://nanopore.usegalaxy.eu).

Workflows

In order to increase the utility of this toolkit, we have developed a set of Galaxy workflows performing common analysis tasks using the tools in the NanoGalaxy toolkit.

Metagenomics taxonomic classification

The base quality of nanopore sequencing reads is constantly improving, making the actual assembly of reads more reliable. Further, the long-reads generated by nanopore sequencing can be used to provide valuable information from metagenomics data, including taxonomic classifications.

Kraken2 is a k-mer based classification technique that can efficiently assign the taxa of long reads that are resilient to the noisy nature of long-read data. The input reads for Kraken2 are compared to a database containing different classes and domains of life that are pre-indexed for algorithm efficiency. Within the NanoGalaxy toolkit we provide a workflow for taxonomic classification using Kraken2, including the post-processing of data and visualization of the results as interactive pie charts using the Krona tool [14].

Nanopolish tutorials

Nanopolish includes an extensive set of software tools for analysing nanopore long-read information at the raw signal level. Further, accompanying Nanopolish documentation provides intuitive tutorials on common scenarios, such as variation analysis and base methylation calling from the raw and mapped signals - Loman et al. [15]. We have integrated Nanopolish and its tutorials into Nanogalaxy in the form of workflows that can be used by researchers to analyse and interpret common quality values for their data.

De novo assembly of genome with highly repetitive repeats

Compared to short reads, long-read data has the advantage of facilitating the assembly of large genomes that contain high numbers of repetitive elements. Schmid et al. utilised Flye and several other tools to generate a comprehensive assembly of the *Pseudomonas koreensis* genome, identifying that the genome has near identical repeat pairs up to 70 kilobase pairs in length [16]. These workflows have also been integrated in the NanoGalaxy toolkit.

Worked example: Antimicrobial resistance

As a further illustration of the utility of the NanoGalaxy toolkit and workflows, we describe below a full end-to-end workflow within Galaxy. This analysis pipeline performs a microbial resistance detection in clinical samples. We describe this workflow in more detail in our training manual on the Galaxy Training materials repository (https://training.galaxyproject.org; Antibiotic resistance detection).

Background

According to the World Health Organization (WHO) and the Organisation for Economic Co-operation and Development (OECD), antimicrobial resistance (AMR) has become one of the biggest threats to global health, food security and economic development [17, 18]. Approximately 50,000 lives per year are lost due to AMR infections within the USA and Europe [19] and AMR infections are expected to increase, reaching 10 million deaths per year by 2050 [20].

Further, the misuse of antibiotics in medical, veterinary and agricultural sectors continues to contribute to the alarming

global rise in antibiotic resistant infections - an increase that may ultimately lead to an era where common infections could once again be lethal. However, the (rapid) detection of antimicrobial resistant pathogens and their resistances in diseases, food and the environment are pillars by which increasing AMR could be detected, monitored and prevented.

Conventional methods for the identification of antimicrobial resistances involves microbial isolation (via culture) and phenotypic typing, which together can take a few days or weeks to complete [21]. Moreover, not all microbial species are amenable to laboratory-based culturing [22]. DNA-sequencing technologies may be utilised to sequence the genomes of cultured microorganisms for the presence of antimicrobial resistance genes, which reduces the time-to-result time. Currently, Illumina sequencing is most widely used, but using this sequencing technology generates difficulties in correctly identifying repetitive insertion sequences, sequences that may flank horizontally acquired genes associated with AMR [23]. Nanopore long-read assemblies could improve resolving these repetitive regions.

Use case 1: Long-read sequencing analysis

The Nanogalaxy toolkit incorporates a rapid long-read assembly workflow employing minimap2 [24], miniasm [25] and Racon [26].Tools for further analysis in the toolkit include Staramr [27] for resistance gene detection, PlasFlow [28] and Bandage [29] for microbial species/plasmid determination and NanoPlot [30] for quality assessment.

In this worked example, the outcome of the Nanogalaxy pipeline was compared to the plasmid sequences recovered by Li et al. [31] (Table S1). The pipeline recovered 19 out of 21 plasmids, with an average identity of 97.76%. The number of detected resistance genes was higher than that found by Li et al. [31], which was expected as Staramr[27] includes the PointFinder (chromosomal point mutations) database [32] and current long-read sequencing may generate relatively high sequence error rates.

Use case 2: Combining short- and long-read sequencing

The previously described long-read assembly workflow rapidly assembles genomes. Since short-read sequencing platforms tend to have a higher accuracy at single-nucleotide level, hybrid solutions to gain from both short- and long-read data are of special interest. The NanoGalaxy toolkit includes a workflow that processes both long- and short- read sequences. In this respect, Unicycler was integrated into the NanoGalaxy toolkit in order to combine the best features of long- and short-sequencing technologies. The workflow recommended by the Unicycler developers [33] includes: Trim Galore [34], Porechop [35] and Filtlong [36] for quality trimming; Unicycler [33] for de novo assembly and bandage [29] for plasmid visualization. These tools are available as stand-alone tools and combined in a NanoGalaxy workflow.

The assembly graphs shown in Fig. 1, compare the NanoGalaxy toolkit with the results from Wick et al. [33]. The Illumina-only (short-read sequencing) graphs show no clear structure(s) present, whereas Nanopore- only (long-read sequencing) is able to generate the circularized structure expected of plasmids. The combination of both sequence techniques gives the clearest view of the circular assemblage expected of plasmids, analogues to the results obtained by Wick et al. [33] (Figure 1). Note that different combinations of short- and long-read tools can be used individually, or combined, to generate personalized workflows.

Conclusion

In this work we covered some important aspects of long-read sequencing analysis with a special focus on ONT sequencing data. We aggregated commonly-used tools into a single consistent inter-operable interface and presented solutions for metagenomic analysis and genome assembly. Furthermore, other long-read sequencing data analysis tools have been developed are currently under development, however we have focused on the most established and widely-used tools. Nevertheless, we expect that the toolkit will be further extended by the community, as NanoGalaxy is part of the open Galaxy platform and Galaxy community. Lastly, the majority of the integrated tools that support other technologies such as PacBio should also work inside Galaxy. However, here we have done an intensive testing of the integrated tools for ONT data.

Methods

Implementation

The tools and workflows included in the NanoGalaxy toolkit enable non-bioinformatics-trained researchers to perform extensive genomics analysis using long-read sequence data, without the need for any coding skills. All tools and their dependencies are installed on the Galaxy platform and are managed by the Conda framework for dependency management. NanoGalaxy tools and their dependencies are available from the Bioconda Conda channel [37]. The Galaxy wrappers are developed openly on GitHub, utilizing the Travis continuous integration framework [38] for testing, and have been made available on the Galaxy ToolShed [13].

Training Materials

An online training manual for the AMR use case described in this publication, as well as a description of NanoGalaxy tools and end-to-end workflows can be found on the Galaxy training materials website [39].

Future Work

The availability of long-read sequencing platforms and data analysis tools is relatively new, with improvements in technology and software continually being developed. As more tools become available these will need to be assembled into existing or new toolkits. Additionally, the future availability of toolkits such as NanoGalaxy will help popularise long-read sequencing, while making it accessible to non-bioinformatics-trained researchers of the future.

Availability of source code and requirements

- Project name: NanoGalaxy
- Project home page: `https://nanopore.usegalaxy.eu`
- Training Manual: `https://training.galaxyproject.org/training-material/topics/metagenomics/tutorials/plasmid-metagenomics-nanopore/tutorial.html`
- License: GNU GPL
- BiotoolsID: nanogalaxy
- RRID: SCR_018912

All developed Galaxy wrappers are available for installation from the Galaxy ToolShed (`https://toolshed.g2.bx.psu.edu/`). The corresponding code repositories for the tool wrappers are

listed in Table 2. The workflows described in this work are publicly available from the European Galaxy server, as well as published Galaxy histories with an example run of each of these workflows (Table 3).

Galaxy Resources

- Galaxy Home Page: https://galaxyproject.org/
- Galaxy Tutorials: https://training.galaxyproject.org
- How to install Galaxy: https://getgalaxy.org
- How to install tools: https://galaxyproject.org/admin/tools/add-tool-from-toolshed-tutorial/
- Full Administrative resources: https://docs.galaxyproject.org/
- Galaxy Help Forum: https://help.galaxyproject.org/
- Connect with the Galaxy Community on Gitter Chat: https://gitter.im/galaxyproject/Lobby/

Availability of supporting data and materials

The data presented here to illustrate the functionality of the tools was obtained from previous publications [40, 31] and was collected and made available from Zenodo https://doi.org/10.5281/zenodo.3741446 [41].

Additional supporting data are available from the GigaScience GigaDB database [42]

Declarations

List of abbreviations

- AMR: Antimicrobial Resistance
- OECD: Organisation for Economic Co-operation and Development
- ONT: Oxford Nanopore Technologies
- SNPs: Single Nucleotide Polymorphisms
- WHO: World Health Organization

Competing Interests

The authors declare that they have no competing interests.

Funding

This project was made possible with the support of Support Casper and the Albert Ludwig University of Freiburg. This project has received funding from the European Union's Horizon 2020 research and innovation programme under grant agreement 825775.

Author's Contributions

WdK, MM and SH contributed to toolkit development and writing of the manuscript. AH tested and evaluated the tools and suggested modifications, feature requests and user improvements. JH contributed to AMR tool and nanopore sequencing discussions and the writing of the manuscript. MvdB and SF contributed to the tool development. BG contributed to the tool development, manuscript writing and supervised the project. DM, RB, and AS supervised the project.

All authors approved the final version of the manuscript.

Acknowledgements

The authors would like to give a special thanks to James Taylor, a leader of the Galaxy Project, and one of its original members who, with great sadness, passed away on April 2, 2020. Furthermore, we would like to thank the Galaxy community for their help in reviewing, testing, and validating the tools presented here.

References

1. Gilissen C, Hoischen A, Brunner HG, Veltman JA. Unlocking Mendelian disease using exome sequencing. Genome biology 2011;12(9):228.
2. de Koning AJ, Gu W, Castoe TA, Batzer MA, Pollock DD. Repetitive elements may comprise over two-thirds of the human genome. PLoS genetics 2011;7(12):e1002384.
3. Goodwin S, McPherson JD, McCombie WR. Coming of age: ten years of next-generation sequencing technologies. Nature Reviews Genetics 2016;17(6):333.
4. Feuk L, Carson AR, Scherer SW. Structural variation in the human genome. Nature Reviews Genetics 2006;7(2):85.
5. Jain M, Olsen HE, Paten B, Akeson M. The Oxford Nanopore MinION: delivery of nanopore sequencing to the genomics community. Genome biology 2016;17(1):239.
6. Rhoads A, Au KF. PacBio sequencing and its applications. Genomics, proteomics & bioinformatics 2015;13(5):278–289.
7. Tsai YC, Greenberg D, Powell J, Hoijer I, Ameur A, Strahl M, et al. Amplification-free, CRISPR-Cas9 targeted enrichment and SMRT sequencing of repeat-expansion disease causative genomic regions. bioRxiv 2017;p. 203919.
8. Flusberg BA, Webster DR, Lee JH, Travers KJ, Olivares EC, Clark TA, et al. Direct detection of DNA methylation during single-molecule, real-time sequencing. Nature methods 2010;7(6):461.
9. Köster J, Rahmann S. Snakemake—a scalable bioinformatics workflow engine. Bioinformatics 2012 08;28(19):2520–2522. https://doi.org/10.1093/bioinformatics/bts480.
10. Di Tommaso P, Chatzou M, Floden EW, Barja PP, Palumbo E, Notredame C. Nextflow enables reproducible computational workflows. Nature biotechnology 2017;35(4):316–319.
11. Afgan E, Baker D, Batut B, van den Beek M, Bouvier D, Čech M, et al. The Galaxy platform for accessible, reproducible and collaborative biomedical analyses: 2018 update. Nucleic Acids Res 2018;46(W1):W537–W544. https://doi.org/10.1093/nar/gky379.
12. Zotero list of Citations of the Galaxy project;. https://www.zotero.org/groups/1732893/galaxy.
13. Galaxy Tool Shed;. https://toolshed.g2.bx.psu.edu/.
14. Ondov BD, Bergman NH, Phillippy AM. Interactive metagenomic visualization in a Web browser. BMC bioinformatics 2011;12(1):385.
15. Loman NJ, Quick J, Simpson JT. A complete bacterial genome assembled de novo using only nanopore sequencing data. Nature methods 2015;12(8):733.
16. Schmid M, Frei D, Patrignani A, Schlapbach R, Frey JE, Remus-Emsermann MN, et al. Pushing the limits of de novo genome assembly for complex prokaryotic genomes harboring very long, near identical repeats. Nucleic acids research 2018;46(17):8953–8965.
17. Organisation for Economic Co-operation and Development, Antimicrobial Resistance; 2017.
18. World Health Organization, Antibiotic resistance; 2018.
19. O'Neill J. Antimicrobial resistance: tackling a crisis for the health and wealth of nations. Review on an-

timicrobial resistance. Review on Antimicrobial Resistance, London, United Kingdom: https://amr-review org/sites/default/files/AMR% 20Review% 20Paper 2014;.
20. O'Neil J, Tackling a crisis for the health and wealth of nations; 2014.
21. Quick J, Ashton P, Calus S, Chatt C, Gossain S, Hawker J, et al. Rapid draft sequencing and real-time nanopore sequencing in a hospital outbreak of Salmonella. Genome Biology 2015 may;16(1):114.
22. Mitsuhashi S, Kryukov K, Nakagawa S, Takeuchi J, Shiraishi Y, Asano K, et al. A portable system for metagenomic analyses using nanopore-based sequencer and laptop computers can realize rapid on-site determination of bacterial compositions. bioRxiv 2017;p. 101865.
23. Ashton PM, Nair S, Dallman T, Rubino S, Rabsch W, Mwaigwisya S, et al. MinION nanopore sequencing identifies the position and structure of a bacterial antibiotic resistance island. Nature Biotechnology 2014 dec;33:296.
24. Li H. Minimap2: pairwise alignment for nucleotide sequences. Bioinformatics 2018 sep;34(18):3094–3100.
25. Li H. Minimap and miniasm: fast mapping and de novo assembly for noisy long sequences. Bioinformatics (Oxford, England) 2016;32(14):2103–10.
26. Vaser R, Sović I, Nagarajan N, Šikić M. Fast and accurate de novo genome assembly from long uncorrected reads. Genome Research 2017;27(5):737–746.
27. Staramr. Github https://github.com/phac-nml/staramr; 2018.
28. Krawczyk PS, Lipinski L, Dziembowski A. PlasFlow: predicting plasmid sequences in metagenomic data using genome signatures. Nucleic acids research 2018 apr;46(6):e35.
29. Wick RR, Schultz MB, Zobel J, Holt KE. Bandage: Interactive visualization of de novo genome assemblies. Bioinformatics 2015 oct;31(20):3350–3352.
30. De Coster W, D'Hert S, Schultz DT, Cruts M, Van Broeckhoven C. NanoPack: visualizing and processing long-read sequencing data. Bioinformatics (Oxford, England) 2018 aug;34(15):2666–2669.
31. Li R, Xie M, Dong N, Lin D, Yang X, Wong MHY, et al. Efficient generation of complete sequences of MDR-encoding plasmids by rapid assembly of MinION barcoding sequencing data. GigaScience 2018;7(3):1–9.
32. Zankari E, Allesøe R, Joensen KG, Cavaco LM, Lund O, Aarestrup FM. PointFinder: a novel web tool for WGS-based detection of antimicrobial resistance associated with chromosomal point mutations in bacterial pathogens. Journal of Antimicrobial Chemotherapy 2017;72(10):2764–2768.
33. Wick RR, Judd LM, Gorrie CL, Holt KE. Unicycler: Resolving bacterial genome assemblies from short and long sequencing reads. PLoS Computational Biology 2017 jun;13(6):e1005595.
34. Kreuger F, Trim Galore! Github https://github.com/FelixKrueger/TrimGalore; 2016.
35. Wick R, Porechop. Github https://github.com/rrwick/Porechop; 2017.
36. Wick R, Filtlong. Github https://github.com/rrwick/Filtlong; 2017.
37. Grüning B, Dale R, Sjödin A, Chapman BA, Rowe J, Tomkins-Tinch CH, et al. Bioconda: sustainable and comprehensive software distribution for the life sciences. Nature methods 2018;15(7):475.
38. Travis CI: Test and Deploy with Confidence;. https://travis-ci.org/.
39. Batut B, Hiltemann S, Bagnacani A, Baker D, Bhardwaj V, Blank C, et al. Community-Driven Data Analysis Training for Biology. Cell Systems 2018 jun;6(6):752–758.e1. https://doi.org/10.1016/j.cels.2018.05.012.
40. Wick RR, Judd LM, Gorrie CL, Holt KE. Completing bacterial genome assemblies with multiplex MinION sequencing. Microbial Genomics 2017;3(10):e000132.
41. NanoGalaxy Zenodo;. https://doi.org/10.5281/zenodo.3529597.
42. de Koning W, Miladi M, Hiltemann S, Heikema A, Hays JP, Flemming S, et al., Supporting data for "NanoGalaxy: Nanopore long-read sequencing data analysis in Galaxy". GigaScience Database; 2020. http://dx.doi.org/10.5524/100795.
43. Kolmogorov M, Yuan J, Lin Y, Pevzner PA. Assembly of long, error-prone reads using repeat graphs. Nature biotechnology 2019;37(5):540.
44. Koren S, Walenz BP, Berlin K, Miller JR, Bergman NH, Phillippy AM. Canu: scalable and accurate long-read assembly via adaptive k-mer weighting and repeat separation. Genome research 2017;27(5):722–736.
45. Ruan J, Li H. Fast and accurate long-read assembly with wtdbg2. BioRxiv 2019;p. 530972.
46. Vaser R, Sović I, Nagarajan N, Šikić M. Fast and accurate de novo genome assembly from long uncorrected reads. Genome research 2017;27(5):737–746.
47. Nurk S, Bankevich A, Antipov D, Gurevich A, Korobeynikov A, Lapidus A, et al. Assembling genomes and mini-metagenomes from highly chimeric reads. In: Annual International Conference on Research in Computational Molecular Biology Springer; 2013. p. 158–170.
48. Oxford Nanopore Technologies ONT, Medaka. GitHub; 2018. https://github.com/nanoporetech/medaka.
49. Sović I, Šikić M, Wilm A, Fenlon SN, Chen S, Nagarajan N. Fast and sensitive mapping of nanopore sequencing reads with GraphMap. Nature communications 2016;7:11307.
50. Oxford Nanopore Technologies ONT, ont_fast5_api. GitHub; 2019. https://github.com/nanoporetech/ont_fast5_api.
51. Loman NJ, Quinlan AR. Poretools: a toolkit for analyzing nanopore sequence data. Bioinformatics 2014;30(23):3399–3401.
52. Walker BJ, Abeel T, Shea T, Priest M, Abouelliel A, Sakthikumar S, et al. Pilon: an integrated tool for comprehensive microbial variant detection and genome assembly improvement. PloS one 2014;9(11):e112963.
53. Krzywinski MI, Schein JE, Birol I, Connors J, Gascoyne R, Horsman D, et al. Circos: An information aesthetic for comparative genomics. Genome Research 2009;http://genome.cshlp.org/content/early/2009/06/15/gr.092759.109.abstract.
54. Wood DE, Lu J, Langmead B. Improved metagenomic analysis with Kraken 2. BioRxiv 2019;p. 762302.

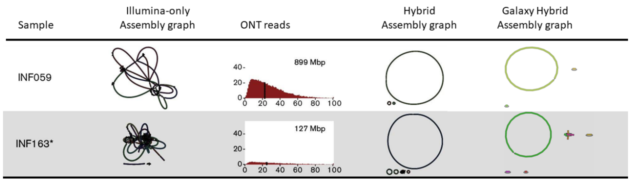

Figure 1. Representation of the output of Wick et al. [33]. The plasmid assembly graphs output created by Bandage [29] are shown to confirm that the workflow works as expected. The length distribution, total yield and N50 of the Oxford Nanopore Technologies (ONT) reads of each *K. pneumoniae* represent the input data.

Table 1. NanoGalaxy toolkit.

Category	Tool name
De novo genome assembly	
	Flye [43]
	Canu [44]
	Unicycler [33]
	Wtdbg2 [45]
	Miniasm [25]
	Racon [46]
	Spades [47]
	Medaka (2 tools) [48]
Long-read mapping	
	Minimap2 [24]
	GraphMap (2 tools) [49]
Polishing, QC and preprocessing	
	ont_fast5_api (4 tools) [50]
	Nanopolish (3 tools) [15]
	Porechop [35]
	Filtlong [36]
	Poretools (13 tools) [51]
	Pilon [52]
Visualization	
	Nanoplot [30]
	Bandage (2 tools) [29]
	Circos [53]
Taxonomy and metagenomics	
	Kraken2 [54]
	PlasFlow [28]
	Staramr [27]
Methylation	
	Nanopolish (1 tool) [15]
Variant calling	
	Medaka (2 tools) [48]

Table 2. Tool availability.

Tool	Github repository
Bandage	https://github.com/galaxyproject/tools-iuc/tree/master/tools/bandage
Canu	https://github.com/bgruening/galaxytools/tree/master/tools/canu
Circos	https://github.com/galaxyproject/tools-iuc/tree/master/tools/circos
Filtlong	https://github.com/galaxyproject/tools-iuc/tree/master/tools/filtlong
Flye	https://github.com/bgruening/galaxytools/tree/master/tools/flye
GraphMap	https://github.com/bgruening/galaxytools/tree/master/tools/graphmap
Kraken2	https://github.com/galaxyproject/tools-iuc/tree/master/tool_collections/kraken2/kraken2
Medaka	https://github.com/galaxyproject/tools-iuc/tree/master/tools/medaka
Miniasm	https://github.com/galaxyproject/tools-iuc/tree/master/tools/miniasm
Minimap2	https://github.com/galaxyproject/tools-iuc/tree/master/tools/minimap2
Nanoplot	https://github.com/galaxyproject/tools-iuc/tree/master/tools/nanoplot
Nanopolish	https://github.com/bgruening/galaxytools/tree/master/tools/nanopolish
NanopolishComp	https://github.com/galaxyproject/tools-iuc/tree/master/tools/nanopolishcomp
Ont_fast5_api	https://github.com/galaxyproject/tools-iuc/tree/master/tools/ont_fast5_api
Pilon	https://github.com/galaxyproject/tools-iuc/tree/master/tools/pilon
PlasFlow	https://github.com/galaxyproject/tools-iuc/tree/master/tools/plasflow
Porechop	https://github.com/galaxyproject/tools-iuc/tree/master/tools/porechop
Poretools	https://github.com/galaxyproject/tools-iuc/tree/master/tools/poretools
Unicycler	https://github.com/galaxyproject/tools-iuc/tree/master/tools/unicycler
Racon	https://github.com/bgruening/galaxytools/tree/master/tools/racon
Spades	https://github.com/galaxyproject/tools-iuc/tree/master/tools/spades
Staramr	https://github.com/phac-nml/galaxy_tools/tree/master/tools/staramr
Wtdbg2	https://github.com/bgruening/galaxytools/tree/master/tools/wtdbg

Table 3. Workflow availability.

Workflow	Link	History	SEEK ID
Basic workflows inspired by the Nanopolish tutorials	https://nanopore.usegalaxy.eu/u/milad/w/nanopolish-variants-tutorial	https://usegalaxy.eu/u/milad/h/nanopolish-tutorial	https://workflowhub.eu/workflows/50?version=1
Genome assembly: Flye-based WF for highly repetitive genomes [Schmid et al. NAR 2018]	https://nanopore.usegalaxy.eu/u/milad/w/ont-assembly-flye-ahrens	https://usegalaxy.eu/u/milad/h/ahrens-nanopore-gm[illegible]	https://workflowhub.eu/workflows/51?version=1
Genome assembly: Unicycler-based WF for Klebsiella pneumoniae [Wick et al. Microbial genomics 2017]	https://usegalaxy.eu/u/milad/h/wick-etal-nanopore	https://usegalaxy.eu/u/milad/h/wick-etal-nanopore	https://workflowhub.eu/workflows/52?version=1
Metagenomics: taxa classification	https://nanopore.usegalaxy.eu/u/milad/w/metagenomics-krakan2	https://usegalaxy.eu/u/milad/h/nanoporebeerdecode[illegible]	https://workflowhub.eu/workflows/53?version=1

Click here to access/download;Manuscript;NanoGalaxy.pdf

GigaScience, 2017, 1–7

doi: xx.xxxx/xxxx
Manuscript in Preparation
Technical Note

TECHNICAL NOTE

NanoGalaxy: Nanopore long-read sequencing data analysis in Galaxy

Willem de Koning[1,2,†*], Milad Miladi[3,†], Saskia Hiltemann[1], Astrid Heikema[4], John P. Hays[4], Stephan Flemming[3], Marius van den Beek[5], Dana A. Mustafa[2], Rolf Backofen[3], Björn Grüning[3,*] and Andrew P. Stubbs[1]

[1]Department of Pathology, Clinical Bioinformatics Unit, Erasmus University Medical Centre, Wytemaweg 80, 3015 CN, Rotterdam, The Netherlands and [2]Department of Pathology, Tumor Immuno-Pathology Laboratory, Erasmus University Medical Centre, ‘s Gravendijkwal 230, 3015 CE, Rotterdam, The Netherlands and [3]Department of Computer Science, Bioinformatics Group, University of Freiburg, 79110 Freiburg im Breisgau, Germany and [4]Department of Medical Microbiology and Infectious Diseases, Erasmus University Medical Centre, ‘s Gravendijkwal 230, 3015 CE, Rotterdam, The Netherlands and [5]Department of Stem Cells and Tissue Homeostasis, Institut Curie, PSL Research University, 75005 Paris, France

[†]Contributed equally.
[*] To whom correspondence should be addressed. w.dekoning.1@erasmusmc.nl; gruening@informatik.uni-freiburg.de

Abstract

Background: Long-read sequencing can be applied to generate very long contigs and even completely assembled genomes at relatively low cost and with minimal sample preparation. As a result, long-read sequencing platforms are becoming more popular. In this respect, the Oxford Nanopore Technologies-based long-read sequencing ’nanopore’ platform is becoming a widely used tool with a broad range of applications and end-users. However, the need to explore and manipulate the complex data generated by long-read sequencing platforms requires accompanying specialised bioinformatics platforms and tools in order to process the long-read data correctly. Importantly, such tools should additionally help democratise bioinformatics analysis by enabling easy access and ease-of-use solutions for researchers.
Results: The Galaxy platform provides a user-friendly interface to computational command-line-based tools, handles the software dependencies and provides refined workflows. The users do not necessarily have to possess programming experience or extended computer skills. The interface enables researchers to perform powerful bioinformatics analysis, including the assembly and analysis of short- or long-read sequence data. The newly developed ‘NanoGalaxy’ is a Galaxy-based toolkit for analysing long-read sequencing data, which is suitable for diverse applications, including de novo genome assembly from genomic, metagenomic and plasmid sequence reads.
Conclusions: A range of best practice tools and workflows for long-read sequence genome assembly has been integrated into a NanoGalaxy platform in order to facilitate easy access and use of bioinformatics tools for researchers. Nanogalaxy is freely available at the European Galaxy server `https://nanopore.usegalaxy.eu` with supporting self-learning training material available at `https://training.galaxyproject.org`.

Key words: Long-read sequencing; Nanopore; Galaxy; Reproducibility; Workflows

Compiled on: September 15, 2020.
Draft manuscript prepared by the author.

Background

Short-read sequencing has become a routine technique within clinical diagnostics [1]. However, the short length of the reads obtained (150-300 bp) complicates the assembly of genomes, especially for highly repetitive regions and the detection of structural variation [2, 3, 4]. Furthermore, even "state-of-the-art" algorithms cannot overcome the issues associated with genome mapping or assembly using short-read sequences. Importantly, advances in sequencing technology now allow *long-read sequencing* to be performed. The two prominent long-read sequencing platforms are nanopore sequencing by Oxford Nanopore Technologies and single molecule real time sequencing by PacBio [5, 6]. These platforms generate sequence reads much longer than the classic short-read technologies, including long-reads from single DNA molecules and without the need of PCR amplification (>10 kilobase on average). Moreover, utilising these technologies, library preparation and sequencing may be performed outside of traditional research laboratories, with sequencing outputs generated in real-time [7]. Protocols that require no PCR amplification also permit the direct detection of base modifications [8].

Analyzing the large amount of data generated by the short- and long-read sequencing technologies is a complex, multi-step process that is computationally intensive and often require bioinformatics expertise. Specifically, for each step in the analysis, a set of different tools or software may be needed. For example, *de novo* assembly is performed via a combination of multiple alignments, assembly and polishing tools, each utilizing its own input parameters. Such tools are typically executed from a UNIX command-line and require extensive computational resources, adding to the complexity of the analysis process. Command-line based workflow managers such as Snakemake and Nextflow [9, 10] can be used for analysing the data. However, these solutions require having expertise in working from the command line. On the other side, some web-based solutions have also been offered. For example EPI2ME platform offers a cloud-based solution with a web interface. The platform supports practical solutions for a limited set of application scenarios, and provides a limited flexibility for configuring the underlying workflows. Here, the Galaxy platforms offers a flexible data analysis platform with a high degree of flexibility, similar to the command-line based workflow managers, and an accessible web interface.

The Galaxy platform reduces the data analysis complexity and implements a standardized and user-friendly interface that accommodates command-line tools and refined workflows complete with their dependencies [11]. The platform hosts a wide range of tools/software and is widely used for bioinformatics analysis within the biological science community [12, 13]. Here we introduce the NanoGalaxy toolkit for analysing Nanopore long-read data. NanoGalaxy comprises a series of integrated Galaxy-based tools that enable researchers to generate powerful short- or long-sequence read assemblies for genomic and plasmid bioinformatics analyses. The NanoGalaxy toolkit is a user-friendly environment that can be utilized inside or outside of traditional research laboratories.

Findings

Tools

We have integrated a large collection of long-read sequence tools into the Galaxy platform, the NanoGalaxy toolkit, including diverse applications for the analyses of long-read sequences (Table 1). This toolkit is freely available from the Galaxy ToolShed, and has additionally been made available as a specialized GalaxyEU subdomain (https://nanopore.usegalaxy.eu).

Workflows

In order to increase the utility of this toolkit, we have developed a set of Galaxy workflows performing common analysis tasks using the tools in the NanoGalaxy toolkit.

Metagenomics taxonomic classification

The base quality of nanopore sequencing reads is constantly improving, making the actual assembly of reads more reliable. Further, the long-reads generated by nanopore sequencing can be used to provide valuable information from metagenomics data, including taxonomic classifications.

Kraken2 is a k-mer based classification technique that can efficiently assign the taxa of long reads that are resilient to the noisy nature of long-read data. The input reads for Kraken2 are compared to a database containing different classes and domains of life that are pre-indexed for algorithm efficiency. Within the NanoGalaxy toolkit we provide a workflow for taxonomic classification using Kraken2, including the post-processing of data and visualization of the results as interactive pie charts using the Krona tool [14].

Nanopolish tutorials

Nanopolish includes an extensive set of software tools for analysing nanopore long-read information at the raw signal level. Further, accompanying Nanopolish documentation provides intuitive tutorials on common scenarios, such as variation analysis and base methylation calling from the raw and mapped signals - Loman et al. [15]. We have integrated Nanopolish and its tutorials into Nanogalaxy in the form of workflows that can be used by researchers to analyse and interpret common quality values for their data.

De novo assembly of genome with highly repetitive repeats

Compared to short reads, long-read data has the advantage of facilitating the assembly of large genomes that contain high numbers of repetitive elements. Schmid et al. utilised Flye and several other tools to generate a comprehensive assembly of the *Pseudomonas koreensis* genome, identifying that the genome has near identical repeat pairs up to 70 kilobase pairs in length [16]. These workflows have also been integrated in the NanoGalaxy toolkit.

Worked example: Antimicrobial resistance

As a further illustration of the utility of the NanoGalaxy toolkit and workflows, we describe below a full end-to-end workflow within Galaxy. This analysis pipeline performs a microbial resistance detection in clinical samples. We describe this workflow in more detail in our training manual on the Galaxy Training materials repository (`https://training.galaxyproject.org`; Antibiotic resistance detection).

Background

According to the World Health Organization (WHO) and the Organisation for Economic Co-operation and Development (OECD), antimicrobial resistance (AMR) has become one of the biggest threats to global health, food security and economic development [17, 18]. Approximately 50,000 lives per year are lost due to AMR infections within the USA and Europe [19] and AMR infections are expected to increase, reaching 10 million deaths per year by 2050 [20].

Further, the misuse of antibiotics in medical, veterinary and agricultural sectors continues to contribute to the alarming

global rise in antibiotic resistant infections - an increase that may ultimately lead to an era where common infections could once again be lethal. However, the (rapid) detection of antimicrobial resistant pathogens and their resistances in diseases, food and the environment are pillars by which increasing AMR could be detected, monitored and prevented.

Conventional methods for the identification of antimicrobial resistances involves microbial isolation (via culture) and phenotypic typing, which together can take a few days or weeks to complete [21]. Moreover, not all microbial species are amenable to laboratory-based culturing [22]. DNA-sequencing technologies may be utilised to sequence the genomes of cultured microorganisms for the presence of antimicrobial resistance genes, which reduces the time-to-result time. Currently, Illumina sequencing is most widely used, but using this sequencing technology generates difficulties in correctly identifying repetitive insertion sequences, sequences that may flank horizontally acquired genes associated with AMR [23]. Nanopore long-read assemblies could improve resolving these repetitive regions.

Use case 1: Long-read sequencing analysis

The Nanogalaxy toolkit incorporates a rapid long-read assembly workflow employing minimap2 [24], miniasm [25] and Racon [26].Tools for further analysis in the toolkit include Staramr [27] for resistance gene detection, PlasFlow [28] and Bandage [29] for microbial species/plasmid determination and NanoPlot [30] for quality assessment.

In this worked example, the outcome of the Nanogalaxy pipeline was compared to the plasmid sequences recovered by Li et al. [31] (Table S1). The pipeline recovered 19 out of 21 plasmids, with an average identity of 97.76%. The number of detected resistance genes was higher than that found by Li et al. [31], which was expected as Staramr[27] includes the PointFinder (chromosomal point mutations) database [32] and current long-read sequencing may generate relatively high sequence error rates.

Use case 2: Combining short- and long-read sequencing

The previously described long-read assembly workflow rapidly assembles genomes. Since short-read sequencing platforms tend to have a higher accuracy at single-nucleotide level, hybrid solutions to gain from both short- and long-read data are of special interest. The NanoGalaxy toolkit includes a workflow that processes both long- and short- read sequences. In this respect, Unicycler was integrated into the NanoGalaxy toolkit in order to combine the best features of long- and short-sequencing technologies. The workflow recommended by the Unicycler developers [33] includes: Trim Galore [34], Porechop [35] and Filtlong [36] for quality trimming; Unicycler [33] for de novo assembly and bandage [29] for plasmid visualization. These tools are available as stand-alone tools and combined in a NanoGalaxy workflow.

The assembly graphs shown in Fig. 1, compare the NanoGalaxy toolkit with the results from Wick et al. [33]. The Illumina-only (short-read sequencing) graphs show no clear structure(s) present, whereas Nanopore- only (long-read sequencing) is able to generate the circularized structure expected of plasmids. The combination of both sequence techniques gives the clearest view of the circular assemblage expected of plasmids, analogues to the results obtained by Wick et al. [33] (Figure 1). Note that different combinations of short- and long-read tools can be used individually, or combined, to generate personalized workflows.

Conclusion

In this work we covered some important aspects of long-read sequencing analysis with a special focus on ONT sequencing data. We aggregated commonly-used tools into a single consistent inter-operable interface and presented solutions for metagenomic analysis and genome assembly. Furthermore, other long-read sequencing data analysis tools have been developed are currently under development, however we have focused on the most established and widely-used tools. Nevertheless, we expect that the toolkit will be further extended by the community, as NanoGalaxy is part of the open Galaxy platform and Galaxy community. Lastly, the majority of the integrated tools that support other technologies such as PacBio should also work inside Galaxy. However, here we have done an intensive testing of the integrated tools for ONT data.

Methods

Implementation

The tools and workflows included in the NanoGalaxy toolkit enable non-bioinformatics-trained researchers to perform extensive genomics analysis using long-read sequence data, without the need for any coding skills. All tools and their dependencies are installed on the Galaxy platform and are managed by the Conda framework for dependency management. NanoGalaxy tools and their dependencies are available from the Bioconda Conda channel [37]. The Galaxy wrappers are developed openly on GitHub, utilizing the Travis continuous integration framework [38] for testing, and have been made available on the Galaxy ToolShed [13].

Training Materials

An online training manual for the AMR use case described in this publication, as well as a description of NanoGalaxy tools and end-to-end workflows can be found on the Galaxy training materials website [39].

Future Work

The availability of long-read sequencing platforms and data analysis tools is relatively new, with improvements in technology and software continually being developed. As more tools become available these will need to be assembled into existing or new toolkits. Additionally, the future availability of toolkits such as NanoGalaxy will help popularise long-read sequencing, while making it accessible to non-bioinformatics-trained researchers of the future.

Availability of source code and requirements

- Project name: NanoGalaxy
- Project home page: `https://nanopore.usegalaxy.eu`
- Training Manual: `https://training.galaxyproject.org/training-material/topics/metagenomics/tutorials/plasmid-metagenomics-nanopore/tutorial.html`
- License: GNU GPL
- BiotoolsID: nanogalaxy
- RRID: SCR_018912

All developed Galaxy wrappers are available for installation from the Galaxy ToolShed (`https://toolshed.g2.bx.psu.edu/`). The corresponding code repositories for the tool wrappers are

listed in Table 2. The workflows described in this work are publicly available from the European Galaxy server, as well as published Galaxy histories with an example run of each of these workflows (Table 3).

Galaxy Resources

- Galaxy Home Page: https://galaxyproject.org/
- Galaxy Tutorials: https://training.galaxyproject.org
- How to install Galaxy: https://getgalaxy.org
- How to install tools: https://galaxyproject.org/admin/tools/add-tool-from-toolshed-tutorial/
- Full Administrative resources: https://docs.galaxyproject.org/
- Galaxy Help Forum: https://help.galaxyproject.org/
- Connect with the Galaxy Community on Gitter Chat: https://gitter.im/galaxyproject/Lobby/

Availability of supporting data and materials

The data presented here to illustrate the functionality of the tools was obtained from previous publications [40, 31] and was collected and made available from Zenodo https://doi.org/10.5281/zenodo.3741446 [41].

Additional supporting data are available from the GigaScience GigaDB database [42]

Declarations

List of abbreviations

- AMR: Antimicrobial Resistance
- OECD: Organisation for Economic Co-operation and Development
- ONT: Oxford Nanopore Technologies
- SNPs: Single Nucleotide Polymorphisms
- WHO: World Health Organization

Competing Interests

The authors declare that they have no competing interests.

Funding

This project was made possible with the support of Support Casper and the Albert Ludwig University of Freiburg. This project has received funding from the European Union's Horizon 2020 research and innovation programme under grant agreement 825775.

Author's Contributions

WdK, MM and SH contributed to toolkit development and writing of the manuscript. AH tested and evaluated the tools and suggested modifications, feature requests and user improvements. JH contributed to AMR tool and nanopore sequencing discussions and the writing of the manuscript. MvdB and SF contributed to the tool development. BG contributed to the tool development, manuscript writing and supervised the project. DM, RB, and AS supervised the project.

All authors approved the final version of the manuscript.

Acknowledgements

The authors would like to give a special thanks to James Taylor, a leader of the Galaxy Project, and one of its original members who, with great sadness, passed away on April 2, 2020. Furthermore, we would like to thank the Galaxy community for their help in reviewing, testing, and validating the tools presented here.

References

1. Gilissen C, Hoischen A, Brunner HG, Veltman JA. Unlocking Mendelian disease using exome sequencing. Genome biology 2011;12(9):228.
2. de Koning AJ, Gu W, Castoe TA, Batzer MA, Pollock DD. Repetitive elements may comprise over two-thirds of the human genome. PLoS genetics 2011;7(12):e1002384.
3. Goodwin S, McPherson JD, McCombie WR. Coming of age: ten years of next-generation sequencing technologies. Nature Reviews Genetics 2016;17(6):333.
4. Feuk L, Carson AR, Scherer SW. Structural variation in the human genome. Nature Reviews Genetics 2006;7(2):85.
5. Jain M, Olsen HE, Paten B, Akeson M. The Oxford Nanopore MinION: delivery of nanopore sequencing to the genomics community. Genome biology 2016;17(1):239.
6. Rhoads A, Au KF. PacBio sequencing and its applications. Genomics, proteomics & bioinformatics 2015;13(5):278–289.
7. Tsai YC, Greenberg D, Powell J, Hoijer I, Ameur A, Strahl M, et al. Amplification-free, CRISPR-Cas9 targeted enrichment and SMRT sequencing of repeat-expansion disease causative genomic regions. bioRxiv 2017;p. 203919.
8. Flusberg BA, Webster DR, Lee JH, Travers KJ, Olivares EC, Clark TA, et al. Direct detection of DNA methylation during single-molecule, real-time sequencing. Nature methods 2010;7(6):461.
9. Köster J, Rahmann S. Snakemake—a scalable bioinformatics workflow engine. Bioinformatics 2012 08;28(19):2520–2522. https://doi.org/10.1093/bioinformatics/bts480.
10. Di Tommaso P, Chatzou M, Floden EW, Barja PP, Palumbo E, Notredame C. Nextflow enables reproducible computational workflows. Nature biotechnology 2017;35(4):316–319.
11. Afgan E, Baker D, Batut B, van den Beek M, Bouvier D, Čech M, et al. The Galaxy platform for accessible, reproducible and collaborative biomedical analyses: 2018 update. Nucleic Acids Res 2018;46(W1):W537–W544. https://doi.org/10.1093/nar/gky379.
12. Zotero list of Citations of the Galaxy project;. https://www.zotero.org/groups/1732893/galaxy.
13. Galaxy Tool Shed;. https://toolshed.g2.bx.psu.edu/.
14. Ondov BD, Bergman NH, Phillippy AM. Interactive metagenomic visualization in a Web browser. BMC bioinformatics 2011;12(1):385.
15. Loman NJ, Quick J, Simpson JT. A complete bacterial genome assembled de novo using only nanopore sequencing data. Nature methods 2015;12(8):733.
16. Schmid M, Frei D, Patrignani A, Schlapbach R, Frey JE, Remus-Emsermann MN, et al. Pushing the limits of de novo genome assembly for complex prokaryotic genomes harboring very long, near identical repeats. Nucleic acids research 2018;46(17):8953–8965.
17. Organisation for Economic Co-operation and Development, Antimicrobial Resistance; 2017.
18. World Health Organization, Antibiotic resistance; 2018.
19. O'Neill J. Antimicrobial resistance: tackling a crisis for the health and wealth of nations. Review on an-

timicrobial resistance. Review on Antimicrobial Resistance, London, United Kingdom: https://amr-review org/sites/default/files/AMR% 20Review% 20Paper 2014;.
20. O'Neil J, Tackling a crisis for the health and wealth of nations; 2014.
21. Quick J, Ashton P, Calus S, Chatt C, Gossain S, Hawker J, et al. Rapid draft sequencing and real-time nanopore sequencing in a hospital outbreak of Salmonella. Genome Biology 2015 may;16(1):114.
22. Mitsuhashi S, Kryukov K, Nakagawa S, Takeuchi J, Shiraishi Y, Asano K, et al. A portable system for metagenomic analyses using nanopore-based sequencer and laptop computers can realize rapid on-site determination of bacterial compositions. bioRxiv 2017;p. 101865.
23. Ashton PM, Nair S, Dallman T, Rubino S, Rabsch W, Mwaigwisya S, et al. MinION nanopore sequencing identifies the position and structure of a bacterial antibiotic resistance island. Nature Biotechnology 2014 dec;33:296.
24. Li H. Minimap2: pairwise alignment for nucleotide sequences. Bioinformatics 2018 sep;34(18):3094–3100.
25. Li H. Minimap and miniasm: fast mapping and de novo assembly for noisy long sequences. Bioinformatics (Oxford, England) 2016;32(14):2103–10.
26. Vaser R, Sović I, Nagarajan N, Šikić M. Fast and accurate de novo genome assembly from long uncorrected reads. Genome Research 2017;27(5):737–746.
27. Staramr. Github https://github.com/phac-nml/staramr; 2018.
28. Krawczyk PS, Lipinski L, Dziembowski A. PlasFlow: predicting plasmid sequences in metagenomic data using genome signatures. Nucleic acids research 2018 apr;46(6):e35.
29. Wick RR, Schultz MB, Zobel J, Holt KE. Bandage: Interactive visualization of de novo genome assemblies. Bioinformatics 2015 oct;31(20):3350–3352.
30. De Coster W, D'Hert S, Schultz DT, Cruts M, Van Broeckhoven C. NanoPack: visualizing and processing long-read sequencing data. Bioinformatics (Oxford, England) 2018 aug;34(15):2666–2669.
31. Li R, Xie M, Dong N, Lin D, Yang X, Wong MHY, et al. Efficient generation of complete sequences of MDR-encoding plasmids by rapid assembly of MinION barcoding sequencing data. GigaScience 2018;7(3):1–9.
32. Zankari E, Allesøe R, Joensen KG, Cavaco LM, Lund O, Aarestrup FM. PointFinder: a novel web tool for WGS-based detection of antimicrobial resistance associated with chromosomal point mutations in bacterial pathogens. Journal of Antimicrobial Chemotherapy 2017;72(10):2764–2768.
33. Wick RR, Judd LM, Gorrie CL, Holt KE. Unicycler: Resolving bacterial genome assemblies from short and long sequencing reads. PLoS Computational Biology 2017 jun;13(6):e1005595.
34. Kreuger F, Trim Galore! Github https://github.com/FelixKrueger/TrimGalore; 2016.
35. Wick R, Porechop. Github https://github.com/rrwick/Porechop; 2017.
36. Wick R, Filtlong. Github https://github.com/rrwick/Filtlong; 2017.
37. Grüning B, Dale R, Sjödin A, Chapman BA, Rowe J, Tomkins-Tinch CH, et al. Bioconda: sustainable and comprehensive software distribution for the life sciences. Nature methods 2018;15(7):475.
38. Travis CI: Test and Deploy with Confidence;. https://travis-ci.org/.
39. Batut B, Hiltemann S, Bagnacani A, Baker D, Bhardwaj V, Blank C, et al. Community-Driven Data Analysis Training for Biology. Cell Systems 2018 jun;6(6):752–758.e1. https://doi.org/10.1016/j.cels.2018.05.012.
40. Wick RR, Judd LM, Gorrie CL, Holt KE. Completing bacterial genome assemblies with multiplex MinION sequencing. Microbial Genomics 2017;3(10):e000132.
41. NanoGalaxy Zenodo;. https://doi.org/10.5281/zenodo.3529597.
42. de Koning W, Miladi M, Hiltemann S, Heikema A, Hays JP, Flemming S, et al., Supporting data for "NanoGalaxy: Nanopore long-read sequencing data analysis in Galaxy". GigaScience Database; 2020. http://dx.doi.org/10.5524/100795.
43. Kolmogorov M, Yuan J, Lin Y, Pevzner PA. Assembly of long, error-prone reads using repeat graphs. Nature biotechnology 2019;37(5):540.
44. Koren S, Walenz BP, Berlin K, Miller JR, Bergman NH, Phillippy AM. Canu: scalable and accurate long-read assembly via adaptive k-mer weighting and repeat separation. Genome research 2017;27(5):722–736.
45. Ruan J, Li H. Fast and accurate long-read assembly with wtdbg2. BioRxiv 2019;p. 530972.
46. Vaser R, Sović I, Nagarajan N, Šikić M. Fast and accurate de novo genome assembly from long uncorrected reads. Genome research 2017;27(5):737–746.
47. Nurk S, Bankevich A, Antipov D, Gurevich A, Korobeynikov A, Lapidus A, et al. Assembling genomes and minimetagenomes from highly chimeric reads. In: Annual International Conference on Research in Computational Molecular Biology Springer; 2013. p. 158–170.
48. Oxford Nanopore Technologies ONT, Medaka. GitHub; 2018. https://github.com/nanoporetech/medaka.
49. Sović I, Šikić M, Wilm A, Fenlon SN, Chen S, Nagarajan N. Fast and sensitive mapping of nanopore sequencing reads with GraphMap. Nature communications 2016;7:11307.
50. Oxford Nanopore Technologies ONT, ont_fast5_api. GitHub; 2019. https://github.com/nanoporetech/ont_fast5_api.
51. Loman NJ, Quinlan AR. Poretools: a toolkit for analyzing nanopore sequence data. Bioinformatics 2014;30(23):3399–3401.
52. Walker BJ, Abeel T, Shea T, Priest M, Abouelliel A, Sakthikumar S, et al. Pilon: an integrated tool for comprehensive microbial variant detection and genome assembly improvement. PloS one 2014;9(11):e112963.
53. Krzywinski MI, Schein JE, Birol I, Connors J, Gascoyne R, Horsman D, et al. Circos: An information aesthetic for comparative genomics. Genome Research 2009;http://genome.cshlp.org/content/early/2009/06/15/gr.092759.109.abstract.
54. Wood DE, Lu J, Langmead B. Improved metagenomic analysis with Kraken 2. BioRxiv 2019;p. 762302.

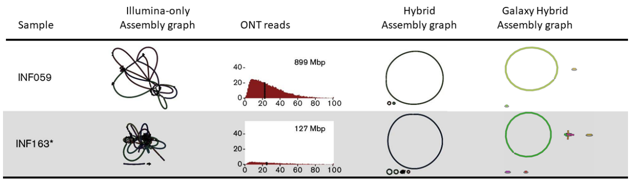

Figure 1. Representation of the output of Wick et al. [33]. The plasmid assembly graphs output created by Bandage [29] are shown to confirm that the workflow works as expected. The length distribution, total yield and N50 of the Oxford Nanopore Technologies (ONT) reads of each *K. pneumoniae* represent the input data.

Table 1. NanoGalaxy toolkit.

Category	Tool name
De novo genome assembly	
	Flye [43]
	Canu [44]
	Unicycler [33]
	Wtdbg2 [45]
	Miniasm [25]
	Racon [46]
	Spades [47]
	Medaka (2 tools) [48]
Long-read mapping	
	Minimap2 [24]
	GraphMap (2 tools) [49]
Polishing, QC and preprocessing	
	ont_fast5_api (4 tools) [50]
	Nanopolish (3 tools) [15]
	Porechop [35]
	Filtlong [36]
	Poretools (13 tools) [51]
	Pilon [52]
Visualization	
	Nanoplot [30]
	Bandage (2 tools) [29]
	Circos [53]
Taxonomy and metagenomics	
	Kraken2 [54]
	PlasFlow [28]
	Staramr [27]
Methylation	
	Nanopolish (1 tool) [15]
Variant calling	
	Medaka (2 tools) [48]

Table 2. Tool availability.

Tool	Github repository
Bandage	https://github.com/galaxyproject/tools-iuc/tree/master/tools/bandage
Canu	https://github.com/bgruening/galaxytools/tree/master/tools/canu
Circos	https://github.com/galaxyproject/tools-iuc/tree/master/tools/circos
Filtlong	https://github.com/galaxyproject/tools-iuc/tree/master/tools/filtlong
Flye	https://github.com/bgruening/galaxytools/tree/master/tools/flye
GraphMap	https://github.com/bgruening/galaxytools/tree/master/tools/graphmap
Kraken2	https://github.com/galaxyproject/tools-iuc/tree/master/tool_collections/kraken2/kraken2
Medaka	https://github.com/galaxyproject/tools-iuc/tree/master/tools/medaka
Miniasm	https://github.com/galaxyproject/tools-iuc/tree/master/tools/miniasm
Minimap2	https://github.com/galaxyproject/tools-iuc/tree/master/tools/minimap2
Nanoplot	https://github.com/galaxyproject/tools-iuc/tree/master/tools/nanoplot
Nanopolish	https://github.com/bgruening/galaxytools/tree/master/tools/nanopolish
NanopolishComp	https://github.com/galaxyproject/tools-iuc/tree/master/tools/nanopolishcomp
Ont_fast5_api	https://github.com/galaxyproject/tools-iuc/tree/master/tools/ont_fast5_api
Pilon	https://github.com/galaxyproject/tools-iuc/tree/master/tools/pilon
PlasFlow	https://github.com/galaxyproject/tools-iuc/tree/master/tools/plasflow
Porechop	https://github.com/galaxyproject/tools-iuc/tree/master/tools/porechop
Poretools	https://github.com/galaxyproject/tools-iuc/tree/master/tools/poretools
Unicycler	https://github.com/galaxyproject/tools-iuc/tree/master/tools/unicycler
Racon	https://github.com/bgruening/galaxytools/tree/master/tools/racon
Spades	https://github.com/galaxyproject/tools-iuc/tree/master/tools/spades
Staramr	https://github.com/phac-nml/galaxy_tools/tree/master/tools/staramr
Wtdbg2	https://github.com/bgruening/galaxytools/tree/master/tools/wtdbg

Table 3. Workflow availability.

Workflow	Link	History	SEEK ID
Basic workflows inspired by the Nanopolish tutorials	https://nanopore.usegalaxy.eu/u/milad/w/nanopolish-variants-tutorial	https://usegalaxy.eu/u/milad/h/nanopolish-tutorial	https://workflowhub.eu/workflows/50?version=1
Genome assembly: Flye-based WF for highly repetitive genomes [Schmid et al. NAR 2018]	https://nanopore.usegalaxy.eu/u/milad/w/ont-assembly-flye-ahrens	https://usegalaxy.eu/u/milad/h/ahrens-nanopore-gm[illegible]	https://workflowhub.eu/workflows/51?version=1
Genome assembly: Unicycler-based WF for Klebsiella pneumoniae [Wick et al. Microbial genomics 2017]	https://usegalaxy.eu/u/milad/h/wick-etal-nanopore	https://usegalaxy.eu/u/milad/h/wick-etal-nanopore	https://workflowhub.eu/workflows/52?version=1
Metagenomics: taxa classification	https://nanopore.usegalaxy.eu/u/milad/w/metagenomics-krakan2	https://usegalaxy.eu/u/milad/h/nanoporebeerdecode[illegible]	https://workflowhub.eu/workflows/53?version=1

Click here to access/download
Supplementary Material
supplementary.tex

Click here to access/download
Supplementary Material
NanoGalaxy Supp.pdf
