# Supplementary material for: NanoGalaxy: Nanopore long-read sequencing data analysis in Galaxy
Source: Gigascience. 2020 Oct 17;9(10):giaa105. doi: 10.1093/gigascience/giaa105 (PMC7568507; doi:10.1093/gigascience/giaa105)
Supplement: giaa105_Supplemental_Files [file giaa105_supplemental_files.zip › NanoGalaxy Supp.pdf]

**Table S1.** The plasmids found by the workflow are BLAST against the plasmids recovered by R. Li et al..

| Barcode | Plasmid | Size | Structural | No. of resistance genes | No. of resistance genes – paper | Coverage | Identity | Comment |
|---|---|---|---|---|---|---|---|---|
| RB01 | RB01-LZ135-CTX-128976 | 128976 | Circular | 8 | 8 | 100 | 98.615 | |
| | RB01-LZ135-NDM-90845 | 90845 | Circular | 10 | 5 | 99 | 98.788 | |
| RB02 | RB02-JN105-IncF-TET-116277-N | 116277 | Circular | 4 | 6 | 99 | 97.872 | |
| | RB02-JN105-IncN-CTX-139496-N | 142307 | Circular | 10 | 9 | 99 | 97.796 | |
| | RB02-JN105-IncN-NDM6-55342 | 55342 | Circular | 11 | 3 | 99 | 86.689 | Not the first hit |
| | RB02-JN105-IncX-NDM5-45823 | 45823 | Circular | 3 | 1 | 99 | 98.449 | |
| | RB02-JN105-IncY-CTX-98443 | 98443 | Circular | 0 | 0 | 99 | 98.771 | |
| RB03 | RB03-WH96T-IncF-OXA-153088 | 153088 | Circular | 5 | 3 | 100 | 98.355 | |
| | RB03-WH96T-IncN-NDM1-56215 | 56215 | Circular | 6 | 2 | 100 | 98.278 | |
| RB04 | RB04-SZ584-1T-IncF-TET-114056 | 114065 | Circular | 6 | 7 | 99 | 98.387 | |
| | RB04-SZ584-1T-IncX3-NDM1-56K-NC | 55919 | Circular | 6 | 2 | 26 | 97.452 | Not the first hit |
| | RB04-SZ584-1T-IncY-130821 | 130821 | Circular | 0 | 0 | 99 | 98.322 | |
| RB05 | RB05-C267-IncA/C-CTX-166467 | 166467 | Circular | 8 | 10 | 99 | 98.92 | |
| RB06 | RB06-C499-IncA/C-CTX-192739 | 192739 | Circular | 11 | 11 | 100 | 98.675 | |
| RB07 | RB07-vb0506-IncA/C-CTX-133742 | 133742 | Circular | 5 | 6 | 100 | 98.348 | |
| RB09 | RB09-IncN-KPC-68571 | 68571 | Circular | 34 | 7 | 100 | 98.497 | |
| RB10 | RB10-29KPC-IncF-TET-136532 | 136532 | Circular | 10 | 12 | 99 | 98.658 | |
| | RB10-29KPC-IncY-KPC-98K-N | 95908 | Circular | 2 | 1 | 99 | 97.769 | |
| RB11 | RB11-IncF-IncHI-KPC-238153 | 238153 | Circular | 2 | 2 | 99 | 98.48 | |
| RB12 | RB12-74T-KPC-IncF-115K-N | 115689 | Circular | 0 | 0 | 99 | 97.948 | |
| | RB12-74T-KPC-IncN-IncX1-KPC-108K-N | 107969 | Circular | 5 | 5 | 100 | 97.927 | |
| **Total / Average** | | | | **146** | **100** | **95.86** | **97.76** | |
