# Supplementary material for: NanoGalaxy: Nanopore long-read sequencing data analysis in Galaxy
Source: Gigascience. 2020 Oct 17;9(10):giaa105. doi: 10.1093/gigascience/giaa105 (PMC7568507; doi:10.1093/gigascience/giaa105)
Supplement: giaa105_Response_to_Reviewer_Comments_Original_Submission [file giaa105_response_to_reviewer_comments_original_submission.pdf]

Author's Response To Reviewer Comments

Close

10/08/2020

Dear Scott,

First of all, I would like to thank you for considering our manuscript "NanoGalaxy: Nanopore long-read sequencing data analysis in Galaxy". NanoGalaxy is now registered in bio.tools and SciCrunch.org. The identifiers can be found in "Availability of source code and requirements". Furthermore, we addressed all points of the reviewers and hope that you'll consider it for publication. We included a point-by-point description in the "Response to Reviewers" box, so the reviewers can easily track the changes made.

Considering the comments by reviewer #3, we think that most of them are beyond the scope of the presented work. Our work introduces the support for analyzing long-read ONT data for the first time in Galaxy. This would let the community to improve and extend on this basis with the vast and booming collection of tools that are getting developed for analyzing long-read data.

All the best,

Willem

Dear Wouter,

Thanks for your review regarding our manuscript. Below you'll find a point-by-point description of the changes made according to your suggestions.

> 1) The selection of tools and training seems to suggest the current toolkit is mostly tailored to the analysis of bacterial data which is a very likely application of the MinION but mentions little about the applications of nanopore sequencing on PromethION in for example structural variant calling in human genetics or assembly of large plant genomes. Are those within the scope of NanoGalaxy and does the platform scale to datasets of that magnitude?

The current set of tools provide a basic set to work with long-read data, especially with ONT data. It is slightly tailored to bacterial data analysis but it can be used in other use cases as well. For example the recently started https://covid19.galaxyproject.org project is using our tools for studying the SARS-CoV-2.

Galaxy as a platform can scale as much as the underlying infrastructure scales. In the case of the European Galaxy server, which we recommend in this manuscript, we have capacity to scale easily to the given use-case. The European Galaxy server is offering 6000 CPUs, 50 TB of memory and 2 PB of storage. It currently serves more than 16.000 researchers and is executing half a million jobs a month.

> 2) The authors mention that single nucleotide polymorphisms cannot be reliably detected due to the higher error rate. However multiple tools have been developed for this purpose (e.g. LongShot) and they perform rather well. This does also conflict with the "nanopolish variants" tutorials.

Indeed, tools performing rather well for SNPs do exist. Therefore, the paragraph of use case 2 about single nucleotide polymorphisms is updated in this revision, to clarify our intention about the advantages of hybrid-solutions without downplaying the capabilities of long-read sequencing.

All the best,

Willem

Dear David,

Thanks for your review regarding our manuscript. Below you'll find a point-by-point description of the changes made according to your comments and suggestions.

> - Findable: usegalaxy repository; findability would be improved by including a persistent DOI for the work. For example, the full workflow(s) at time of review submission could be added to the existing NanoGalaxy workflow repository (which seems to currently only include input fastq files), or on protocols.io, or hosted in GigaDB.

We improved the findability by including the workflows to workflowhub.eu and adding their SEEK ID to the workflow availability table.

> I did attempt to load some of my own nanopore data from SRA [SRR4734731], but the first stage [Download and Extract Reads in FASTA/Q format from NCBI SRA] produced an error, "Failed to write job script

The loading of your Nanopore data seems to work fine by now. At the time of reviewing there might have been server problems at the Galaxy sight, which caused the error to occur. The history including the loaded data and a workflow run are available at https://usegalaxy.eu/u/willem/h/srr4734731-nanogalaxy.

> The tool appears to have been tested (with example results from one workflow demonstrated in the paper), but *is not* compared to existing tools / methods for processing of nanopore data. Given that the presented tool incorporates existing methods in an easier-to-use interface, I can accept that a comparison is not strictly necessary, although a mention of bioinformatics workflows that exist within the nanopore community would be helpful (and, for example, explaining why those workflows may not be appropriate for public analysis of nanopore data).

All tools have been reviewed and tested separately. In addition the integration of tool tests into a Continuous Integration system ensures that the tools will also work with future updates. NanoGalaxy is offering a toolset with which 100s of workflows can be built.

Other Nanopore workflows like the ones from NF-core do exist and are as appropriate to use as workflows developed in NanoGalaxy. However, the Galaxy platform focuses on tools and ensures with a lot of tests that tools are working and can be freely combined. This provides users the possibility and freedom to create new workflows in a graphical way without having Unix- or Command Line skills. In this way NanoGalaxy is unique and enables the creation of hundreds of workflows in an easy, robust and scalable way. If tools are the basic building blocks, it is also very easy to combine NanoGalaxy tools with other tools like Circos, numerous post-processing tools or virology tools.

For this revision, we have extended the introduction and explained the advantages of Galaxy over alternative platforms for accessible analysis of nanopore data.

The tool shows innovation in the approach of nanopore data analysis by aggregating commonly-used tools into a single consistent interoperable interface, which is a greatly-appreciated need in this area.

> The tool shows innovation in the approach of nanopore data analysis by aggregating commonly-used tools into a single consistent interoperable interface, which is a greatly-appreciated need in this area.

We now reflect this in the conclusion. Thanks!

> Perhaps in light of the passing of James Taylor this year, the authors could acknowledge the contribution that he has made in managing the Galaxy project. A citation where Galaxy is first mentioned in the introduction would also be a good idea [e.g. DOI: 10.1101/gr.4086505].

The acknowledgement suggestion is indeed a great way to thank James Taylor for the work and is added.

All the best,
Willem

Dear Federico,

Thanks for your review regarding our manuscript. Below you'll find a point-by-point description of the changes made according to your suggestions.

> -My first point is about the focus of the toolkit on ONT rather than, more in general, long-read technologies. In all the manuscript, there is no hint that other long-read sequencing technologies do exist, in particular, of course, I am pointing the authors at PacBio.

We added a sentence about the other long-read sequencing platform to the introduction.

> Most of the tools in the toolkit can work with reads from both technologies. So, why restrict the focus to ONT? If there is some rationale for this that I am not getting, please explain it in the manuscript since other readers may incur the same bafflement.

We acknowledge that most of the tools should work on other long-read-sequencing technologies, but they're only tested intensively on ONT. Therefore, it is presented as Nanopore long-read sequencing data analysis.This point is mentioned within the conclusion section in this revision.

> The second point is about the relatively small number of tools in the toolkit. I am not necessarily a fan of having 1000 tools for the same task if they are selected with a careful eye, but in its current implementation, Nanogalaxy simply leaves out many of the possible applications of long-read sequencing technologies. In detail (I am giving a few examples of available tools for each application, this does not mean that I consider them the best for the task nor the ones that should be included in the toolkit, they are just a pointer in the right direction):

Our work introduces the support for analyzing long-read ONT data for the first time in Galaxy and introduces the technical basis to extend the Galaxy platform with long-read tools. We explicitly see this project as a community starting point and encourage the community to improve and extend on this basis with the vast and booming collection of tools that are getting developed for analyzing long-read data. While the broad range of tools being mentioned is interesting, it goes beyond the scope of this work. In the conclusion part we also reflect on the tool categories provided. See below for a few explanations that do not fit into the paper.

> -- Base-calling

Base-calling of the raw sequencing data is unfortunately excluded from NanoGalaxy. Due to the licensing issues, the state-of-the-art base-calling algorithms could have not been offered to the end-users of the public platforms such as the European Galaxy server."

> -- Structural variation visualization

Circos is added to the manuscript.

> -- Detecting base modifications

There are so many modification tools under development, however we have focused on the most established and widely-used tools (Nanopolish). However, the NanoGalaxy project has just started and we are constantly integrating new tools and observing the field to provide the best experience to our users.

> -- Long read transcriptomes

> -- Detection of short tandem repeats expansion

> -- Long read simulators

Long read simulators are commonly used by researchers who are experts in bioinformatics analysis and benchmarking. According to our experience,such tools would not have a considerable benefit the broader spectrum of Galaxy users.

> - In general, when only one or very few tools are chosen among many for any task or application, an explanation of the rationale behind that choice should be available to the reader. Are these tools the best for their task according to some public benchmark? Are they the most suitable to be integrated into Galaxy? Is there any comparable/better tool that, for any reason (e.g., licensing), could not be included in the toolkit? Random choice?

A rationale behind the choices is given in the conclusion:
“Furthermore, many long-read sequencing tools are currently under development, however we have focused on the most established and widely-used tools. Nevertheless, we expect that the toolkit will be further extended by the community, as NanoGalaxy is part of the open Galaxy platform and Galaxy community.”

> -Table S1 is named Table 1 in the caption

Table 1 is now Table S1 in the caption.

> -The term "Structural variation" should be preferred over "structural variance". Variance is an unrelated, mathematical term.

Thanks a lot, we changed “Structural variance” to “Structural variation”.

> - I am very fond of Galaxy as well as the authors, but I find it unnecessary to report the current number of citations of this platform, that by the way, is destined to change by the time of publication.

Indeed, the current number of citations is removed from the manuscript.

> - The bibliography references are in a bit of a mess. For example, the numbering is not consecutive (e.g., 29 comes after 9). I think this may depend on Table 1, but more attention is needed.

We are sorry about this, but can not spot this error. However, we double checked and will also be taken care of during roof-reading.

> - I am not sure that [7] is the best paper to support the point of bioinformatics expertise since it seems to be more about the application of bioinformatics to the discovery of antibiotic resistance.

Thanks, we agreed to your comment and have removed the citation.

> - The concept of "more comprehensive algorithms" is not completely clear to me. Also, one or more references should be provided for problems with mapping and assembly using short reads.

Thanks, comprehensive is changed to state-of-the-art.

> - high repeats -> highly repetitive or high number/frequency of repeats

Thanks, we changed “High repeats” to “highly repetitive repeats”

> - In the Background section of the worked example, the last paragraph repeats concepts that have already been discussed.

Thanks, indeed it was repetitive and a part is removed.

> - (3) should be (Table 3) in the Availability of source section.

Thanks, (3) is changed to (Table 3) in the Availability of source section.

We hope that the explanations above are sufficient to answer your questions.

All the best,

Willem

Close
