# Supplementary material for: NanoGalaxy: Nanopore long-read sequencing data analysis in Galaxy
Source: Gigascience. 2020 Oct 17;9(10):giaa105. doi: 10.1093/gigascience/giaa105 (PMC7568507; doi:10.1093/gigascience/giaa105)
Supplement: giaa105_Response_to_Reviewer_Comments_Revision_1 [file giaa105_response_to_reviewer_comments_revision_1.pdf]

Author's Response To Reviewer Comments

Close

Dear Scott,

I have added the DOI and reference in the availability of supporting data section.

All the best,
Willem

Dear Scott,

I would like to thank you for considering our manuscript “NanoGalaxy: Nanopore long-read sequencing data analysis in Ga

Furthermore, I sent an email to the curator with the information he asked for.
Hopefully this is sufficient to finalize the manuscript. Otherwise, please let me know.

All the best,
Willem

Dear David,

Thanks for your new review regarding our manuscript. Below you’ll find a point-by-point description of the changes made

Overall summary: I'm happy that most of the issues I raised about the manuscript have been dealt with, and am okay wit
their feet wet using NanoGalaxy. Thank you for giving me an opportunity to explore NanoGalaxy.

I’m glad you appreciate the opportunity to explore NanoGalaxy and want to thank you for recommending the publication.

As far as I can tell, the primary publication for Galaxy (Afgan et al., 2018) is not directly cited, nor is the original 2005 pa
https://eur01.safelinks.protection.outlook.com/?url=https%3A%2F%2Fgalaxyproject.org%2Fciting-galaxy%2F%23primar
publication&data=02%7C01%7Cw.dekoning.1%40erasmusmc.nl%7Cb1868667c5924c1ef0f108d84d7320cd%7C526638ba
introduction. According to that page, the citation for Galaxy Toolshed [reference #12 in the manuscript] should be Blanke

Thanks again for pointing out this issue, and expanding the explanation. Indeed, we added the citation on the suggested l

-- Software / Pipeline evaluation --
Now that I can load datasets in NanoGalaxy, I had another go at using NanoGalaxy blindly with other datasets:
1) used 'fasterq-dump' to retrieve reads from SRR12395855
2) Changed dataset type to 'fastq.gz'
3) Ran 'NanoPlot' on dataset collection 'Single-end data (fasterq-dump)'
4) Run 'Flye Assembly' with estimated genome size of 5m [tool error "cannot find 'ext'"]
5) Run 'Canu assembler' with estimated genome size of 5m
[Stopped after waiting ~16h for job completion]
[I didn't run miniasm, because I didn't know at this stage how to do an all-vs-all mapping via NanoGalaxy]
1) used 'fasterq-dump' to retrieve reads from SRR4734731
[error: "accession 'SRR4734731' is PACBIO, please use fastq-dump instead"]
2) used 'fastq-dump' to retrieve reads from SRR4734731
3) Run 'Canu assembler' with an estimated genome size of 16k.
[Tried to set stopOnLowCoverage=0, but this option wouldn't be accepted]
On further reflection, it would help if assembly tools would give a warning (especially for datasets > 100 Mb) about the tir

Thank you for trying our tools, we have added the following note to the assembly tools based on your feedback: "NOTE: T

Given troubles with my own datasets, I tried to find / test out the "Use Case 1: Long Read Sequencing Analysis" workflow
materials website,
https://eur01.safelinks.protection.outlook.com/?url=https%3A%2F%2Ftraining.galaxyproject.org%2F&data=02%7C01%7
and eventually found (after searching for "nanopore") a Galaxy Training workflow with the title "Antibiotic resistance detec
It would be helpful if a direct link to this workflow were added to the paper, or at the very least a reference to the workflo
https://eur01.safelinks.protection.outlook.com/?url=https%3A%2F%2Ftraining.galaxyproject.org%2Ftraining-material%2
nanopore%2Ftutorial.html&data=02%7C01%7Cw.dekoning.1%40erasmusmc.nl%7Cb1868667c5924c1ef0f108d84d7320c

Indeed the Galaxy training website is mentioned, but not the name of the related training. Therefore, the name of the trai

After accessing and reading this workflow, I was able to run through the example successfully and carry out all operations
* running nanoplot
* running all-vs-all minimap2 on the collected dataset
* generating an assembly with miniasm
* converting the resultant GFA to FASTA format
* remapping reads to the assembled genomes with minimap2
* consensus calling with Racon
* visualising miniasm assembly graph with Bandage
* Assembling using unicycler with nanopore-only reads
Unfortunately, I couldn't find 'PlasFlow' or 'staramr' in the Tools search to attempt those steps. It would be a good idea to

The metagenomics analysis tools indeed seemed to be missing on the website, therefore this category is added to nanopo

Summary: NanoGalaxy is at least loading input datasets, and some tools work. Importantly, the tools in the specified wor

We hope that our changes improve the manuscript as expected.

All the best,
Willem

Close
