# Supplementary material for: NanoGalaxy: Nanopore long-read sequencing data analysis in Galaxy
Source: Gigascience. 2020 Oct 17;9(10):giaa105. doi: 10.1093/gigascience/giaa105 (PMC7568507; doi:10.1093/gigascience/giaa105)
Supplement: giaa105_Reviewer_2_Report_Original_Submission — David Eccles -- 5/10/2020 Reviewed [file giaa105_reviewer_2_report_original_submission.pdf]

Reviewer Report

Title: NanoGalaxy: Nanopore long-read sequencing data analysis in Galaxy

Version: Original Submission **Date:** 5/10/2020

Reviewer name: David Eccles

Reviewer Comments to Author:

This Technical Note discusses an addition to the workflows available on Galaxy: nanopore long-read sequencing data analysis. The presented workflows seem reasonable, and are a needed addition to the public knowledge about nanopore data analysis, but I haven't been able to test them out (presumably due to my own computer setup).
[https://academic.oup.com/gigascience/pages/technical_note]
Technical notes require that reporting guidelines follow Findable, Accessible, Interoperable, Reusable (FAIR) standards. The majority of these are fulfilled by simply presenting an open-source software tool. I've explicity described one aspect of the presented workflow that fulfils these recommendations (note: this is not an exhaustive list of the workflow or recommendations):
- Findable: usegalaxy repository; findability would be improved by including a persistent DOI for the work. For example, the full workflow(s) at time of review submission could be added to the existing NanoGalaxy workflow repository (which seems to currently only include input fastq files), or on protocols.io, or hosted in GigaDB.
- Accessible: public usegalaxy workflow / project homepage (i.e. nanopore.usegalaxy.eu)
- Interoperable: a prerequisite for a tool to work with Galaxy
- Reusable: CC-BY-4.0 license attached to Zenodo repository; CC0-1.0 license mentioned on galaxy project website - "All content on this site is available under CC0-1.0, unless otherwise specified."
I did attempt to load some of my own nanopore data from SRA [SRR4734731], but the first stage [Download and Extract Reads in FASTA/Q format from NCBI SRA] produced an error, "Failed to write job script '/data/dnb02/galaxy_db/job_working_directory/008/200/8200695/tool_script.sh', could not verify job script integrity." This error was essentially identical when uploading the same reads from my local computer, "Failed to write job script '/data/dnb02/galaxy_db/job_working_directory/008/200/8200724/tool_script.sh', could not verify job script integrity.". Unfortunately this means that I can't really evaluate any of the software workflows, so my review is restricted to statements made in the manuscript. Given that the technical note guidelines state that "Submissions that do not meet these requirements will be rejected without review", I'm assuming that this workflow has worked for GigaScience editors, and this is an issue peculiar to my own circumstances.
Criteria for Technical Notes:
1. This manuscript presents a procedure for the analysis or handling of large-scale data.
2. This manuscript describes a tool optimized to run in a cloud environment (i.e. Galaxy).
3. This manuscript describes a new tool. I was made aware of NanoGalaxy through attendance at London Calling, but believe this to be the first publication about the tool.

4. The tool appears to have been tested (with example results from one workflow demonstrated in the paper), but *is not* compared to existing tools / methods for processing of nanopore data. Given that the presented tool incorporates existing methods in an easier-to-use interface, I can accept that a comparison is not strictly necessary, although a mention of bioinformatics workflows that exist within the nanopore community would be helpful (and, for example, explaining why those workflows may not be appropriate for public analysis of nanopore data).
5. The tool shows innovation in the approach of nanopore data analysis by aggregating commonly-used tools into a single consistent interoperable interface, which is a greatly-appreciated neede in this arena.
6. Authors clearly acknowledge the work upon which they are building by indluding references and repositories for the tools that are included in this Galaxy project.
Acknowledgements:
Perhaps in light of the passing of James Taylor this year, the authors could acknowledge the contribution that he has made in managing the Galaxy project. A citation where Galaxy is first mentioned in the introduction would also be a good idea [e.g. DOI: 10.1101/gr.4086505].
[see https://galaxyproject.org/jxtx/]

Level of Interest

Please indicate how interesting you found the manuscript: Choose an item.

Quality of Written English

Please indicate the quality of language in the manuscript: Choose an item.

Declaration of Competing Interests

Please complete a declaration of competing interests, considering the following questions:

- Have you in the past five years received reimbursements, fees, funding, or salary from an organisation that may in any way gain or lose financially from the publication of this manuscript, either now or in the future?
- Do you hold any stocks or shares in an organisation that may in any way gain or lose financially from the publication of this manuscript, either now or in the future?
- Do you hold or are you currently applying for any patents relating to the content of the manuscript?
- Have you received reimbursements, fees, funding, or salary from an organization that holds or has applied for patents relating to the content of the manuscript?
- Do you have any other financial competing interests?
- Do you have any non-financial competing interests in relation to this paper?

If you can answer no to all of the above, write 'I declare that I have no competing interests' below. If your reply is yes to any, please give details below.

I have received free supplies, workshop training, and conference attendance from Oxford Nanopore Technologies in recognition of my contribution to the Nanopore community.

I agree to the open peer review policy of the journal. I understand that my name will be included on my report to the authors and, if the manuscript is accepted for publication, my named report including any attachments I upload will be posted on the website along with the authors' responses. I agree for my report to be made available under an Open Access Creative Commons CC-BY license (http://creativecommons.org/licenses/by/4.0/). I understand that any comments which I do not wish to be included in my named report can be included as confidential comments to the editors, which will not be published.

Choose an item.

To further support our reviewers, we have joined with Publons, where you can gain additional credit to further highlight your hard work (see: https://publons.com/journal/530/gigascience). On publication of this paper, your review will be automatically added to Publons, you can then choose whether or not to claim your Publons credit. I understand this statement.

Yes Choose an item.
