# Supplementary material for: NanoGalaxy: Nanopore long-read sequencing data analysis in Galaxy
Source: Gigascience. 2020 Oct 17;9(10):giaa105. doi: 10.1093/gigascience/giaa105 (PMC7568507; doi:10.1093/gigascience/giaa105)
Supplement: giaa105_Reviewer_2_Report_Revision_1 — David Eccles -- 8/30/2020 Reviewed [file giaa105_reviewer_2_report_revision_1.pdf]

Reviewer Report

Title: NanoGalaxy: Nanopore long-read sequencing data analysis in Galaxy

Version: Revision 1 **Date:** 8/30/2020

Reviewer name: David Eccles

Reviewer Comments to Author:

Overall summary: I'm happy that most of the issues I raised about the manuscript have been dealt with, and am okay with recommending publication as-is, given that additional issues I have are relatively minor. While there are issues with some tools, that is expected from an initial release, and I expect that an suitably inquisitive reader will still be able to get their feet wet using NanoGalaxy.
Thank you for giving me an opportunity to explore NanoGalaxy.
Here's an expansion / clarification of a comment from my first review:
As far as I can tell, the primary publication for Galaxy (Afgan et al., 2018) is not directly cited, nor is the original 2005 paper cited (although on reflection, the 2018 paper would be a better citation). It would be a good idea to include a citation specific to Galaxy, as recommmended in the NanoGalaxy 'How to Cite Galaxy' information:
https://galaxyproject.org/citing-galaxy/#primary-publication
The Galaxy citation would be best in the first sentence of the last paragraph of the introduction.
According to that page, the citation for Galaxy Toolshed [reference #12 in the manuscript] should be Blankenberg et al., 2014.
-- Software / Pipeline evaluation --
Now that I can load datasets in NanoGalaxy, I had another go at using NanoGalaxy blindly with other datasets:
1) used 'fasterq-dump' to retrieve reads from SRR12395855
2) Changed dataset type to 'fastq.gz'
3) Ran 'NanoPlot' on dataset collection 'Single-end data (fasterq-dump)'
4) Run 'Flye Assembly' with estimated genome size of 5m [tool error "cannot find 'ext'"]
5) Run 'Canu assembler' with estimated genome size of 5m
  [Stopped after waiting ~16h for job completion]
[I didn't run miniasm, because I didn't know at this stage how to do an all-vs-all mapping via NanoGalaxy]
1) used 'fasterq-dump' to retrieve reads from SRR4734731
[error: "accession 'SRR4734731' is PACBIO, please use fastq-dump instead"]
2) used 'fastq-dump' to retrieve reads from SRR4734731
3) Run 'Canu assembler' with estimated genome size of 16k.
  Tried to set stopOnLowCoverage=0, but this option wouldn't be accepted
On further reflection, it would help if assembly tools would give a warning (especially for datasets > 100 Mb) about the time taken for the assembly step.
Given troubles with my own datasets, I tried to find / test out the "Use Case 1: Long Read Sequencing

Analysis" workflow. Unfortunately, the manuscript doesn't include information about input datasets. It mentions "In this worked example", but I can't see a worked example anywhere. Earlier in the manuscript, I see a reference to the Galaxy training materials website, https://training.galaxyproject.org, and eventually found (after searching for "nanopore") a Galaxy Training workflow with the title "Antibiotic resistance detection", with authors matching the authors of
this manuscript. That title doesn't seem to be anywhere in the manuscript.
It would be helpful if a direct link to this workflow were added to the paper, or at the very least a reference to the workflow title, "Antibiotic resistance detection":
https://training.galaxyproject.org/training-material/topics/metagenomics/tutorials/plasmid-metagenomics-nanopore/tutorial.html
After accessing and reading this workflow, I was able to run through the example
successfully and carry out all operations:
* running nanoplot
* running all-vs-all minimap2 on the collected dataset
* generating an assembly with miniasm
* converting the resultant GFA to FASTA format
* remapping reads to the assembled genomes with minimap2
* consensus calling with Racon
* visualising miniasm assembly graph with Bandage
* Assembling using unicycler with nanopore-only reads
Unfortunately, I couldn't find 'PlasFlow' or 'staramr' in the Tools search to attempt those steps. It would be a good idea to either add these to NanoGalaxy (in a way that they're visible by search), or create an alternative workflow that stops at the assembly step.
Summary: NanoGalaxy is at least loading input datasets, and some tools work. Importantly, the tools in the specified workflow are working for generating a full assembly, when using the parameters stated in the workflow. I expect that the inconsistency with toolsets will improve over time as more tool paths get more eyes and more use.

Level of Interest

Please indicate how interesting you found the manuscript: Choose an item.

Quality of Written English

Please indicate the quality of language in the manuscript: Choose an item.

Declaration of Competing Interests

Please complete a declaration of competing interests, considering the following questions:

- Have you in the past five years received reimbursements, fees, funding, or salary from an organisation that may in any way gain or lose financially from the publication of this manuscript, either now or in the future?
- Do you hold any stocks or shares in an organisation that may in any way gain or lose financially from the publication of this manuscript, either now or in the future?
- Do you hold or are you currently applying for any patents relating to the content of the manuscript?
- Have you received reimbursements, fees, funding, or salary from an organization that holds or has applied for patents relating to the content of the manuscript?
- Do you have any other financial competing interests?
- Do you have any non-financial competing interests in relation to this paper?

If you can answer no to all of the above, write 'I declare that I have no competing interests' below. If your reply is yes to any, please give details below.

I have received flow cell and reagent discounts from Oxford Nanopore, and participated as a demonstrator / tutor in a few training courses for Nanopore sequencing.

I agree to the open peer review policy of the journal. I understand that my name will be included on my report to the authors and, if the manuscript is accepted for publication, my named report including any attachments I upload will be posted on the website along with the authors' responses. I agree for my report to be made available under an Open Access Creative Commons CC-BY license (http://creativecommons.org/licenses/by/4.0/). I understand that any comments which I do not wish to be included in my named report can be included as confidential comments to the editors, which will not be published.

Choose an item.

To further support our reviewers, we have joined with Publons, where you can gain additional credit to further highlight your hard work (see: https://publons.com/journal/530/gigascience). On publication of this paper, your review will be automatically added to Publons, you can then choose whether or not to claim your Publons credit. I understand this statement.

Yes Choose an item.
