# Supplementary material for: NanoGalaxy: Nanopore long-read sequencing data analysis in Galaxy
Source: Gigascience. 2020 Oct 17;9(10):giaa105. doi: 10.1093/gigascience/giaa105 (PMC7568507; doi:10.1093/gigascience/giaa105)
Supplement: giaa105_Reviewer_3_Report_Original_Submission — Federico Zambelli -- 5/12/2020 Reviewed [file giaa105_reviewer_3_report_original_submission.pdf]

Reviewer Report

Title: NanoGalaxy: Nanopore long-read sequencing data analysis in Galaxy

Version: Original Submission **Date:** 5/12/2020

Reviewer name: Federico Zambelli

Reviewer Comments to Author:

Koning and colleagues present NanoGalaxy, a Galaxy "toolkit" tailored for the analysis of Oxford Technologies (ONT) long reads. The purpose of collecting, organizing, and, above all, making available through Galaxy useful tools for the analysis of this relatively novel type of sequencing data is a commendable and interesting one. However, in its current state, the toolkit seems to focus, without any apparent reason, just on ONT reads, while providing support only for a relatively limited set of tools, covering only a few of the many applications of long-read technologies.
More in detail:
Major:
- My first point is about the focus of the toolkit on ONT rather than, more in general, long-read technologies. In all the manuscript, there is no hint that other long-read sequencing technologies do exist, in particular, of course, I am pointing the authors at PacBio. Most of the tools in the toolkit can work with reads from both technologies. So, why restricting the focus to ONT? If there is some rationale for this that I am not getting, please explain it in the manuscript since other readers may incur the same bafflement.
-The second point is about the relatively small number of tools in the toolkit. I am not necessarily a fan of having 1000 tools for the same task if they are selected with a careful eye, but in its current implementation, Nanogalaxy simply leaves out many of the possible applications of long-read sequencing technologies. In detail (I am giving a few examples of available tools for each application, this does not mean that I consider them the best for the task nor the ones that should be included in the toolkit, they are just a pointer in the right direction):
--Base-calling: base calling of SMRT sequencing is a very active area of research as new approaches become available over time. For example, it is not unusual to reprocess old raw data with new or improved versions of base callers in order to correct errors (e.g., homopolymer accuracy) or to detect modifications that could not be detected before (e.g., Guppy and its relatives, Chiron).
--Structural variation visualization: structural variants detection is one of the most useful applications of long reads sequencing, effectively removing the technical barrier to their study posed by short reads. Many visualization tools are currently available for their representation (see https://doi.org/10.1038/s10038-019-0687-0 for a review).
-- Detecting base modifications: de novo approaches do exist (e.g., Tombo, NanoMod) as well as pre-trained models: Nanopolish (detects 5mC) is included in the toolkit, but many other methods do exist to detect, e.g., RNA m6A (EpiNano), 6mA and 5mC (mCaller, DeepSignal, DeepMod, ...) that are not.
--Long read transcriptomes: long read isoforms detection tools (e.g., Iso-con, SQANTI, TALON) are absent from the toolkit.

--Detection of short tandem repeats expansion: see https://doi.org/10.1093/bib/bbz099 for a review.
--Long read simulators (e.g., SimLoRD, DeepSimulator).
-In general, when only one or very few tools are chosen among many for any task or application, an explanation of the rationale behind that choice should be available to the reader. Are these tools the best for their task according to some public benchmark? Are they the most suitable to be integrated into Galaxy? Is there any comparable/better tool that, for any reason (e.g., licensing), could not be included in the toolkit? Random choice?
Minor:
-Table S1 is named Table 1 in the caption
-The term "Structural variation" should be preferred over "structural variance". Variance is an unrelated, mathematical term.
- I am very fond of Galaxy as well as the authors, but I find it unnecessary to report the current number of citations of this platform, that by the way, is destined to change by the time of publication.
- The bibliography references are in a bit of a mess. For example, the numbering is not consecutive (e.g., 29 comes after 9). I think this may depend on Table 1, but more attention is needed.
- I am not sure that [7] is the best paper to support the point of bioinformatics expertise since it seems to be more about the application of bioinformatics to the discovery of antibiotic resistance.
- The concept of "more comprehensive algorithms" is not completely clear to me. Also, one or more references should be provided for problems with mapping and assembly using short reads.
- high repeats -> highly repetitive or high number/frequency of repeats
- In the Background section of the worked example, the last paragraph repeats concepts that have already been discussed.
- (3) should be (Table 3) in the Availability of source section.

Level of Interest

Please indicate how interesting you found the manuscript: Choose an item.

Quality of Written English

Please indicate the quality of language in the manuscript: Choose an item.

Declaration of Competing Interests

Please complete a declaration of competing interests, considering the following questions:

- Have you in the past five years received reimbursements, fees, funding, or salary from an organisation that may in any way gain or lose financially from the publication of this manuscript, either now or in the future?
- Do you hold any stocks or shares in an organisation that may in any way gain or lose financially from the publication of this manuscript, either now or in the future?

- Do you hold or are you currently applying for any patents relating to the content of the manuscript?
- Have you received reimbursements, fees, funding, or salary from an organization that holds or has applied for patents relating to the content of the manuscript?
- Do you have any other financial competing interests?
- Do you have any non-financial competing interests in relation to this paper?

If you can answer no to all of the above, write 'I declare that I have no competing interests' below. If your reply is yes to any, please give details below.

I declare that I have no competing interests

I agree to the open peer review policy of the journal. I understand that my name will be included on my report to the authors and, if the manuscript is accepted for publication, my named report including any attachments I upload will be posted on the website along with the authors' responses. I agree for my report to be made available under an Open Access Creative Commons CC-BY license (http://creativecommons.org/licenses/by/4.0/). I understand that any comments which I do not wish to be included in my named report can be included as confidential comments to the editors, which will not be published.

Choose an item.

To further support our reviewers, we have joined with Publons, where you can gain additional credit to further highlight your hard work (see: https://publons.com/journal/530/gigascience). On publication of this paper, your review will be automatically added to Publons, you can then choose whether or not to claim your Publons credit. I understand this statement.

Yes Choose an item.
